# Disproportionation of Inorganic Sulfur Compounds by Mesophilic Chemolithoautotrophic *Campylobacterota*

**DOI:** 10.1128/msystems.00954-22

**Published:** 2022-12-21

**Authors:** Shasha Wang, Lijing Jiang, Shaobin Xie, Karine Alain, Zhaodi Wang, Jun Wang, Delin Liu, Zongze Shao

**Affiliations:** a Key Laboratory of Marine Genetic Resources, Third Institute of Oceanography, Ministry of Natural Resources of China, Sino-French Laboratory of Deep-Sea Microbiology (MicrobSea), Xiamen, People’s Republic of China; b State Key Laboratory Breeding Base of Marine Genetic Resources, Sino-French Laboratory of Deep-Sea Microbiology (MicrobSea), Xiamen, People’s Republic of China; c Fujian Key Laboratory of Marine Genetic Resources, Sino-French Laboratory of Deep-Sea Microbiology (MicrobSea), Xiamen, People’s Republic of China; d Southern Marine Science and Engineering Guangdong Laboratory (Zhuhai), Zhuhai, People’s Republic of China; e CNRS, Univ Brest, Ifremer, Unité Biologie et Ecologie des Ecosystèmes Marins Profonds BEEP, UMR 6197, IRP 1211 MicrobSea, IUEM, Plouzané, France; University of Pretoria

**Keywords:** sulfur disproportionation, *Campylobacterota*, hydrothermal vent, chemolithoautotroph

## Abstract

The disproportionation of inorganic sulfur compounds could be widespread in natural habitats, and microorganisms could produce energy to support primary productivity through this catabolism. However, the microorganisms that carry this process out and the catabolic pathways at work remain relatively unstudied. Here, we investigated the bacterial diversity involved in sulfur disproportionation in hydrothermal plumes from Carlsberg Ridge in the northwestern Indian Ocean by enrichment cultures. A bacterial community analysis revealed that bacteria of the genera *Sulfurimonas* and *Sulfurovum*, belonging to the phylum *Campylobacterota* and previously having been characterized as chemolithoautotrophic sulfur oxidizers, were the most dominant members in six enrichment cultures. Subsequent bacterial isolation and physiological studies confirmed that five *Sulfurimonas* and *Sulfurovum* isolates could disproportionate thiosulfate and elemental sulfur. The ability to disproportionate sulfur was also demonstrated in several strains of *Sulfurimonas* and *Sulfurovum* that were isolated from hydrothermal vents or other natural environments. Dialysis membrane experiments showed that S^0^ disproportionation did not require the direct contact of cells with bulk sulfur. A comparative genomic analysis showed that *Campylobacterota* strains did not contain some genes of the Dsr and rDSR pathways (*aprAB*, *dsrAB*, *dsrC*, *dsrMKJOP*, and *qmoABC*) that are involved in sulfur disproportionation in some other taxa, suggesting the existence of an unrevealed catabolic pathway for sulfur disproportionation. These findings provide evidence for the catabolic versatility of these *Campylobacterota* genera, which are widely distributed in chemosynthetic environments, and expand our knowledge of the microbial taxa involved in this reaction of the biogeochemical sulfur cycle in hydrothermal vent environments.

**IMPORTANCE** The phylum *Campylobacterota*, notably represented by the genera *Sulfurimonas* and *Sulfurovum*, is ubiquitous and predominant in deep-sea hydrothermal systems. It is well-known to be the major chemolithoautotrophic sulfur-oxidizing group in these habitats. Herein, we show that the mesophilic predominant chemolithoautotrophs of the genera *Sulfurimonas* and *Sulfurovum* could grow via sulfur disproportionation to gain energy. This is the first report of the chemolithoautotrophic disproportionation of thiosulfate and elemental sulfur within the genera *Sulfurimonas* and *Sulfurovum*, and this comes in addition to their already known role in the chemolithoautotrophic oxidation of sulfur compounds. Sulfur disproportionation via chemolithoautotrophic *Campylobacterota* may represent a previously unrecognized primary production process in hydrothermal vent ecosystems.

## INTRODUCTION

Sulfur is one of the most abundant elements in nature and plays crucial roles in biogeochemical cycling and biomass accumulation (1). Reduced sulfur compounds, such as sulfides, are present in a variety of natural habitats, including freshwater and marine systems, and they are particularly abundant in oxygen minimum zones (OMZ) and deep-sea hydrothermal vents, whereas sulfur predominates in its most oxidized state (i.e., sulfate) in marine systems (2). Between these most extreme reduced and oxidized states, there is a range of intermediate sulfur species (e.g., elemental sulfur, thiosulfate, and sulfite), which are generally present in lower concentrations (2). The oxidation and reduction of sulfur by microorganisms have been well-studied over the past decades in many habitats, including sediments and hydrothermal vent systems (3). Increasing knowledge suggests that sulfur disproportionation may be a nonnegligible microbial catabolism that can support primary productivity in modern sedimentary, aquatic, and hydrothermal environments and can thus play a notable role in the global biogeochemical cycling of carbon, nitrogen, sulfur, and metals (4).

The disproportionation (also known as dismutation) of inorganic sulfur compounds (ISC) is a chemolithotrophic microbial process in which elemental sulfur, thiosulfate, and sulfite serve as both electron donors and acceptors and are also converted to hydrogen sulfide and sulfate (5). This appears to be an ancient mode of microbial energy catabolism that has presumably left significant isotopic signatures in the geological record of sulfur rocks (6). Under standard conditions, the reactions of the disproportionation of thiosulfate and sulfite are exergonic (with a ΔG^0^′ value of −22.3 and −58.9 kJ mol^−1^ of substrate, respectively), whereas the disproportionation of elemental sulfur is endergonic (ΔG^0^′ = 0.3 kJ mol^−1^) (7, 8). However, the Gibbs energy yield of the reaction becomes exergonic under low concentrations of hydrogen sulfide, which is achieved in the environment via the precipitation of sulfide with iron or via rapid oxidation (9).

Microorganisms capable of disproportionating intermediate sulfur species are widespread in a variety of anoxic environments, including marine sediments, soda lakes, and freshwater basins, and they are partly responsible for the isotopic signatures of sulfide minerals in young and old sediments (10). To date, 43 bacterial species in total have been described as being able to disproportionate inorganic sulfur compounds; They belong principally to the phyla *Desulfobacterota* and *Proteobacteria* (11). A few other taxa belonging to the phyla *Firmicutes* (Dethiobacter alkaliphilus and three *Desulfotomaculaceae*) (11), *Nitrospirota* (Dissulfurispira thermophila) (12), and *Campylobacterota* (Desulfurella amilsii) have also been reported (13, 14). In addition, another study analyzing metagenomes from an elemental sulfur Arctic glacial deposit, another form of extreme environment, also suggested that *Campylobacterota* may be involved in sulfur disproportionation (15). Microbially-mediated thiosulfate disproportionation could be an important process in certain environments, such as marine and freshwater anoxic sediments, as revealed by radioisotope tracing (16). The disproportionation of elemental sulfur could date as far back as 3.5 Ga and could be one of the earliest modes of microbial catabolism (17, 18), but this hypothesis is controversial (19).

The metabolic pathways of ISC disproportionation and the microorganisms producing energy by means of this reaction are still poorly documented in hydrothermal vent ecosystems. Until now, only four sulfur-disproportionating bacteria (SDB) have been isolated from deep-sea hydrothermal vent environments (namely, Thermosulfuriphilus ammonigenes, Thermosulfurimonas dismutans, *Thermosulfurimonas* sp. F29, and Dissulfuribacter thermophilus of the phylum *Desulfobacterota*), and two others have been isolated from shallow hydrothermal vents (Dissulfurirhabdus thermomarina and Thermosulfurimonas marina), all of which are thermophilic (20–25). In addition, sulfur disproportionation is also a key process mediated by the uncultivated bacterium “*Candidatus* Desulfobulbus rimicarensis” as a symbiont of the vent shrimp Rimicaris exoculata (26). Recently, calculations of the Gibbs energy of sulfur disproportionation using internally consistent thermodynamic properties and geochemical data from four different hydrothermal systems showed that S^0^ disproportionation is sufficiently exergonic to allow for growth in most niches of hydrothermal ecosystems, regardless of the geological and geochemical context and depth (27). Therefore, sulfur disproportionation may play an irreplaceable role in the biogeochemical cycle of deep-sea hydrothermal vent ecosystems. However, due to the lack of genetic markers specific to this process and the difficulty of interpreting the *in situ* isotopic data of sulfur species, the contribution of sulfur disproportionation in deep-sea hydrothermal systems remains unknown to date.

Hydrothermal plumes are one of the major components of hydrothermal vent fields in the global midocean ridge system, and they have a profound influence on elemental cycles in the ocean (28). Hydrothermal plumes represent an extended mixed gradient between the venting fluid and the seawater, allowing for the settlement of luxuriant microbial and animal communities that derive their energy via chemosynthesis through oxidation-reduction reactions (29, 30). The disproportionation of sulfur compounds in hydrothermal plumes has not been studied because the identification of sulfur-disproportionating bacteria relies on either cultural approaches or activity measurements. As such, we hypothesized here that sulfur disproportionation occurs in plumes, and we tested this hypothesis. We isolated pure cultures representing the predominant members within sulfur-disproportionating enrichment cultures of hydrothermal plumes of the Carlsberg Ridge in the northwest Indian Ocean, and we tested their activity for sulfur disproportionation. Furthermore, the interactions between the cells and S^0^ were analyzed, and a comparative genomic analysis was carried out. These results will expand our knowledge of the sulfur cycle and will improve our understanding of the life strategies and ecological roles of some vent microorganisms.

## RESULTS

### Enrichment cultures of SDB from deep-sea hydrothermal plume samples.

Nine plume samples were enriched with elemental sulfur as the sole substrate and ferrihydrite as a sulfide scavenger, and they were incubated at 28°C in a resting incubator. After 3 rounds of successive transfer to a fresh liquid medium, 6 positive cultures of bacterial consortia were obtained after 20 to 40 days of incubation. H_2_S production was observed in all of the positive cultures and was visible by the color change when ferrihydrite chelated the produced H_2_S and formed black ferrous sulfide minerals (Fig. S1A). Concomitantly, sulfate, the other end product of sulfur disproportionation, was also detected and was observed to have accumulated in the concentration range of 2.24 to 8.50 mM (Fig. S1B). In addition, bacterial growth over time also confirmed that sulfur disproportionation was mediated by bacterial activity. Under a fluorescent light microscope, all of the enrichment cultures were teeming with living bacterial cells (Fig. S2). In contrast, the uninoculated negative control, after its subcultures in fresh medium, showed no generation of sulfide and sulfate. These results indicate that bacteria possessing the capacity to grow by sulfur disproportionation are common in this deep-sea hydrothermal vent plume.

10.1128/msystems.00954-22.1 FIG S1 Visual aspects after culture growth of six positive consortia obtained under S^0^ disproportionation conditions (A) and the corresponding sulfate concentrations (B). Download FIG S1, TIF file, 1.4 MB. Copyright © 2022 Wang et al. 2022 Wang et al. https://creativecommons.org/licenses/by/4.0/ This content is distributed under the terms of the Creative Commons Attribution 4.0 International license.

10.1128/msystems.00954-22.2 FIG S2 Viability of bacterial cells in culture (third subcultures) and in a negative control after two weeks of incubation. Live cells are shown in green, and dead cells are shown in red. Download FIG S2, TIF file, 2.4 MB. Copyright © 2022 Wang et al. 2022 Wang et al. https://creativecommons.org/licenses/by/4.0/ This content is distributed under the terms of the Creative Commons Attribution 4.0 International license.

### Bacterial composition of the SDB consortia, as revealed by Illumina sequencing.

To reveal the composition of the bacterial communities involved in S^0^ disproportionation in the vent plumes, the six SDB consortia were subjected to a bacterial diversity analysis via high-throughput sequencing. After filtering the raw reads and removing low-quality reads, 45,212 to 58,601 effective sequences were collected from each sample, resulting in a total of 302,821 sequences (Table S1). The high-quality sequences were grouped into 151 amplicon sequence variants (ASVs) at a similarity level of 100%. The representative sequences of 151 ASVs and their distribution across the samples are shown in Table S3. Bacterial α-diversity analyses showed that the bacterial species richness (Chao1) values of the samples ST0104, ST0121, and ST0258 were higher than those of the other SDB communities (Table S1). The diversity of prokaryotes, as calculated by the Shannon index, varied over a wide range from moderate values (0.2) to relatively high values (>2.0) (Table S1). Sample ST0121 possessed the highest bacterial diversity value, whereas the bacterial diversity values of samples ST0126 and ST0116 were the lowest (Table S1).

10.1128/msystems.00954-22.6 TABLE S1 Number of ASVs and alpha diversity indices for microbial communities. Download Table S1, DOCX file, 0.01 MB. Copyright © 2022 Wang et al. 2022 Wang et al. https://creativecommons.org/licenses/by/4.0/ This content is distributed under the terms of the Creative Commons Attribution 4.0 International license.

10.1128/msystems.00954-22.8 TABLE S3 The representative sequences of ASVs and their distribution across the samples. Download Table S3, XLS file, 0.2 MB. Copyright © 2022 Wang et al. 2022 Wang et al. https://creativecommons.org/licenses/by/4.0/ This content is distributed under the terms of the Creative Commons Attribution 4.0 International license.

**FIG 1:**
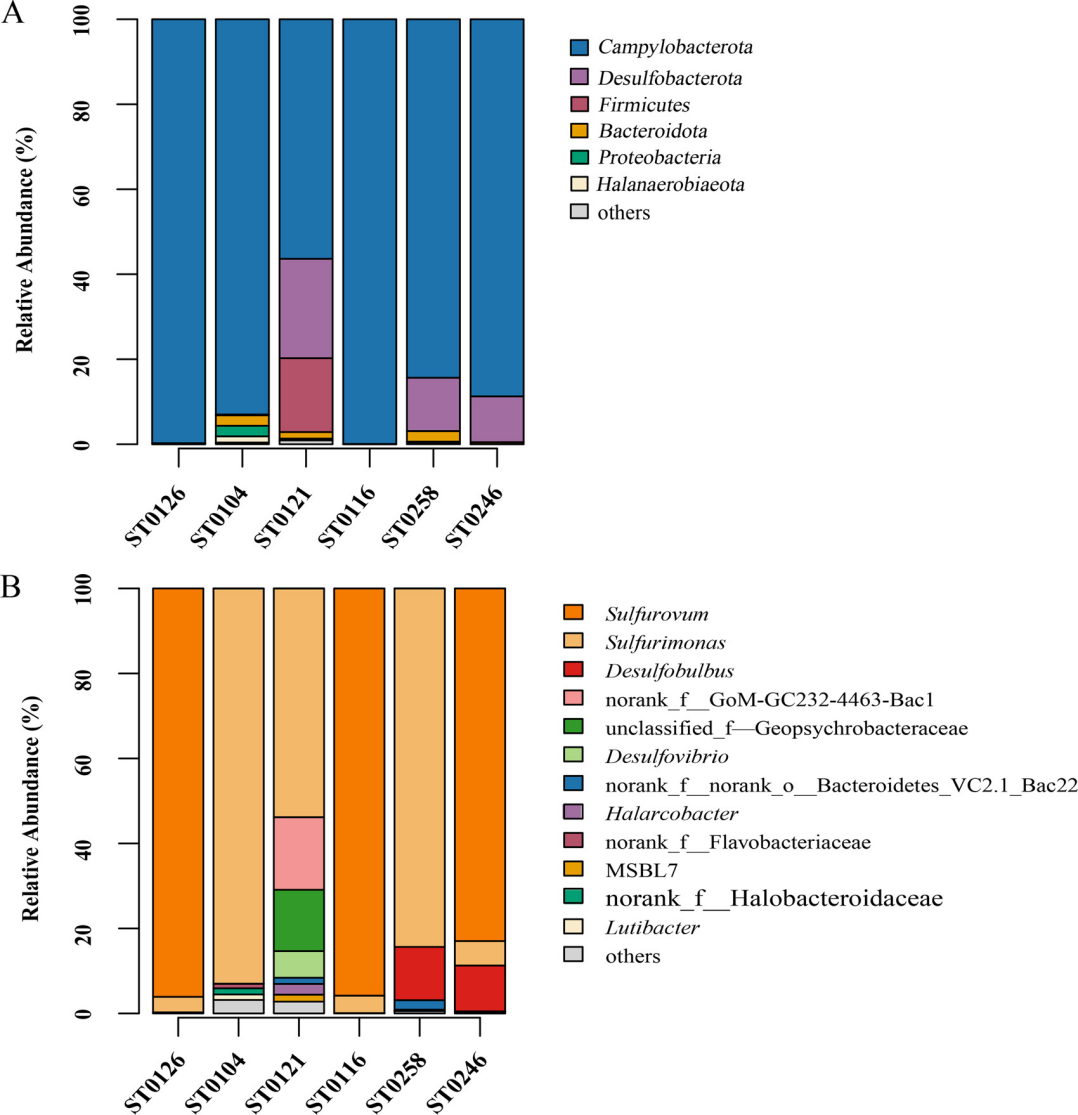
Taxonomic assignments and relative abundances of sequences at the phylum (A) and genus (B) level of six SDB consortia from vent plumes. For each plot, all taxa present in a relative abundance of ≥1.0% in at least one of the samples are shown, whereas those present in <1.0% relative abundance are grouped with unassigned sequences in the “others” category.

The microbial compositions of the bacterial communities grown under S^0^ disproportionation conditions are shown in Fig. 1. At the phylum level, *Campylobacterota* was the most predominant phylum in the six consortia, accounting for 56.4 to 99.9% of the total relative abundance of bacteria, followed by the phyla *Desulfobacterota* (10.8 to 23.4%), *Firmicutes* (0.2 to 17.4%), *Bacteroidota* (0.2 to 2.5%), and *Proteobacteria* (0.4 to 2.5%) (Fig. 1A). Among the *Campylobacterota*, the class *Campylobacteria* was predominant in all of the plume samples and was mainly composed of representatives of the genera *Sulfurimonas* and *Sulfurovum*. However, these genera were distributed differently in the different samples. Representatives of the genus *Sulfurimonas* were present in all of the cultivated consortia, accounting for 3.7 to 93.0% of the relative abundance of total sequences. They predominated in the consortia from samples ST0104, ST0121, and ST0258 (Fig. 1B). Representatives of the genus *Sulfurovum* were only present and dominant in samples ST0116, ST0126, and ST0246, accounting for 83.0 to 96.1% of the relative abundance of total sequences (Fig. 1B). In addition, representatives of the phylum *Desulfobacterota* also accounted for a fairly large fraction of the diversity in some consortia obtained in culture. For example, sequences belonging to the genus *Desulfobulbus*, a genus for which the ability to disproportionate sulfur has already been reported (10), occurred in samples ST0246 and ST0258, accounting for 10.8% and 12.5% of all sequences, respectively (Fig. 1B). Sequences affiliated with the genus *Desulfovibrio*, which also encompasses sulfur disproportionators (11), were found in sample ST0121, accounting for 6.28% of the bacterial sequences (Fig. 1B).

### Isolation of chemoautotrophic SDB from the cultivated consortia.

**FIG 2:**
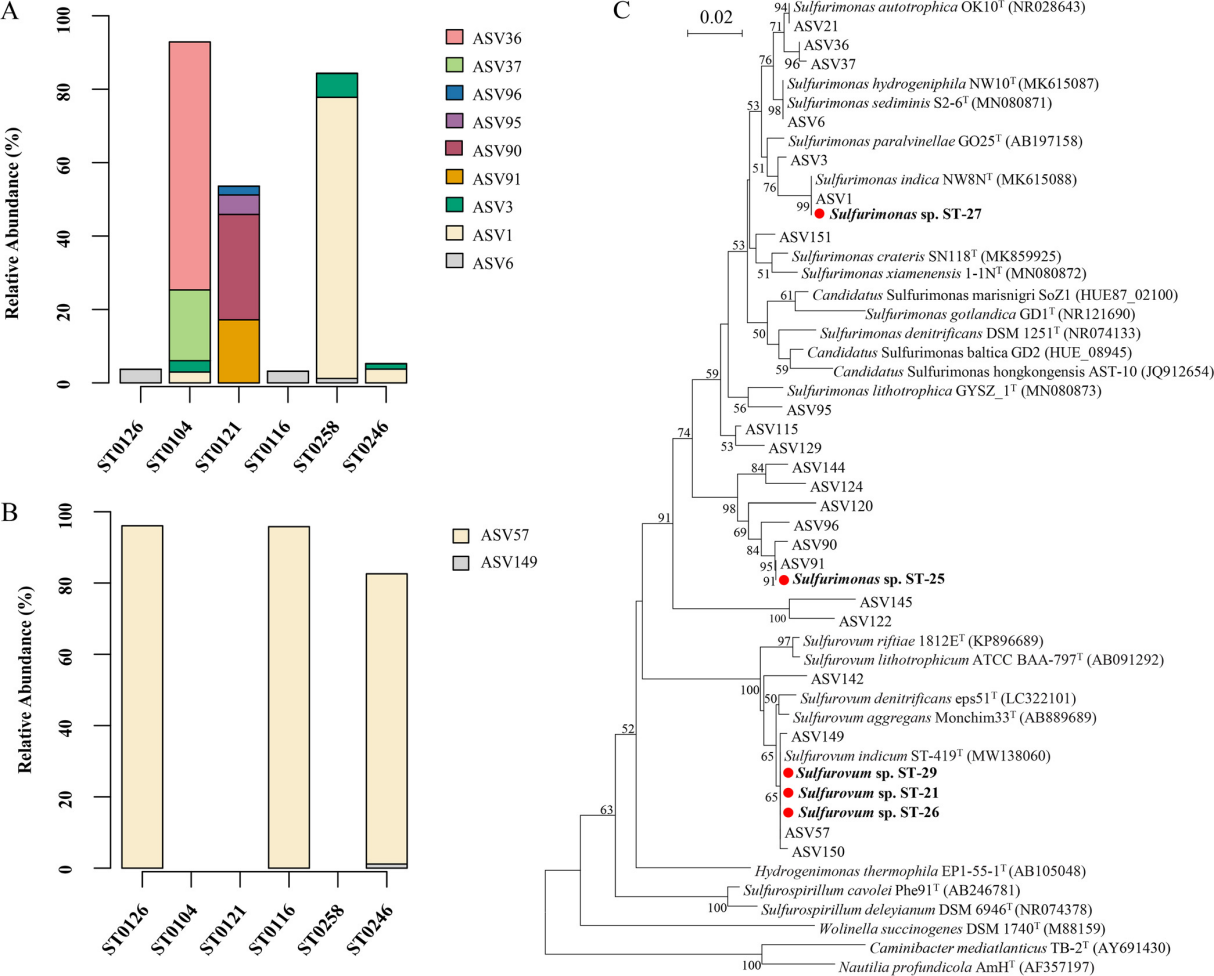
*Sulfurimonas* and *Sulfurovum* distribution within cultivated consortia and a phylogenetic analysis of the 16S rRNA gene phylotypes. (A and B) Distribution of *Sulfurimonas* (A) and *Sulfurovum* (B) ASVs in the different SDB consortia. Only ASVs representing >1% of the ASVs in at least one sample are shown. (C) Phylogenetic tree of the ASVs and the isolate sequences of *Sulfurimonas* and *Sulfurovum* identified in this study. The tree was constructed using MEGA 6.0 with the neighbor-joining (NJ) method, based on representative ASV sequences, the 16S rRNA gene sequences of the isolates, and type strains. Each scale bar represents 0.02 nucleotide changes per position.

Surprisingly, chemolithoautotrophic bacteria of the genera *Sulfurimonas* and *Sulfurovum*, which are known for their ability to oxidize sulfur, represented the predominant taxa in consortia from vent plume samples that were grown under S^0^ disproportionation conditions. To confirm their ability to perform sulfur disproportionation, bacteria were isolated and purified from the consortia. Five bacterial strains were obtained through dilution-extinction series from the six cultivated consortia (Table 1). Phylogenetic analyses of their 16S rRNA gene sequences located them within the genera *Sulfurimonas* and *Sulfurovum*. Among them, two isolates belonged to the genus *Sulfurimonas*, and these were referenced as strains *Sulfurimonas* sp. ST-25 and *Sulfurimonas* sp. ST-27. Strain ST-25, which shared 93.3% homology with the complete 16S rRNA sequence of *Sulfurimonas lithotrophica* GYSZ_1^T^, likely represents a new genomic species or even a new genus (31). Strain ST-27 was most closely related to *Sulfurimonas indica* NW8N^T^, displaying 99.6% sequence similarity (Table 1). A total of 18 ASVs belonging to the genus *Sulfurimonas* were detected in all consortia, with ASV36, ASV1, and ASV90 being the most predominant ones in samples ST0104, ST0258, and ST0121 and accounting for 67.5%, 76.6%, and 28.7% the of total bacterial sequences, respectively (Fig. 2A). The phylogenetic tree constructed from the sequences of ASVs, isolates, and reference type strains (Fig. 2C) showed that strains ST-25 and ST-27 corresponded to the predominant ASV91 (with 17.2% abundance) and ASV1 in the enrichment cultures, respectively (Fig. 2C). Thus, with the exception of ASV1, most of the dominant members belonging to the genus *Sulfurimonas* in the sulfur disproportionation consortia need to be further isolated.

**TABLE 1:** Strains isolated from the SDB consortia from a hydrothermal plume and the disproportionation abilities or inabilities for different sulfur compounds

Isolates	Sampling sites	Sulfur disproportionation^*a*^			Most closely related type strain	16 rRNA gene sequence similarity (%)
		Elemental sulfur	Thiosulfate	Sulfite		
ST-21	ST0126	+	+	−	*Sulfurovum indicum* ST-419^T^	98.92
ST-25	ST0121	++	++	−	*Sulfurimonas lithotrophica* GYSZ_1^T^	93.34
ST-26	ST0116	+	+	−	*Sulfurovum indicum* ST-419^T^	98.95
ST-27	ST0258	++	++	−	*Sulfurimonas indica* NW8N^T^	99.62
ST-29	ST0246	++	++	−	*Sulfurovum indicum* ST-419^T^	99.91

a ++, strongly positive; +, positive; −, negative.

Moreover, three *Sulfurovum* strains, namely, ST-21, ST-26, and ST-29 were isolated from the consortia ST0126, ST0116, and ST0246. They shared the highest homology to *Sulfurovum indicum* ST-419^T^, displaying 98.9%, 99.0%, and 99.9% sequence similarity, respectively, based on full-length 16S rRNA gene sequences (Table 1). Furthermore, 4 ASVs belonging to the genus *Sulfurovum* were detected, and ASV57 was predominant in samples ST0116, ST0126, and ST0246, displaying 81.4 to 96.1% relative abundance (Fig. 2B). The phylogenetic analysis showed that the three isolates formed a cluster with ASV57 and *Sulfurovum indicum* ST-419^T^ (Fig. 2C). These results suggest that the predominant members of the genus *Sulfurovum* that are involved in sulfur disproportionation were isolated.

### Sulfur disproportionation in isolates of *Sulfurimonas* and *Sulfurovum*.

**FIG 3:**
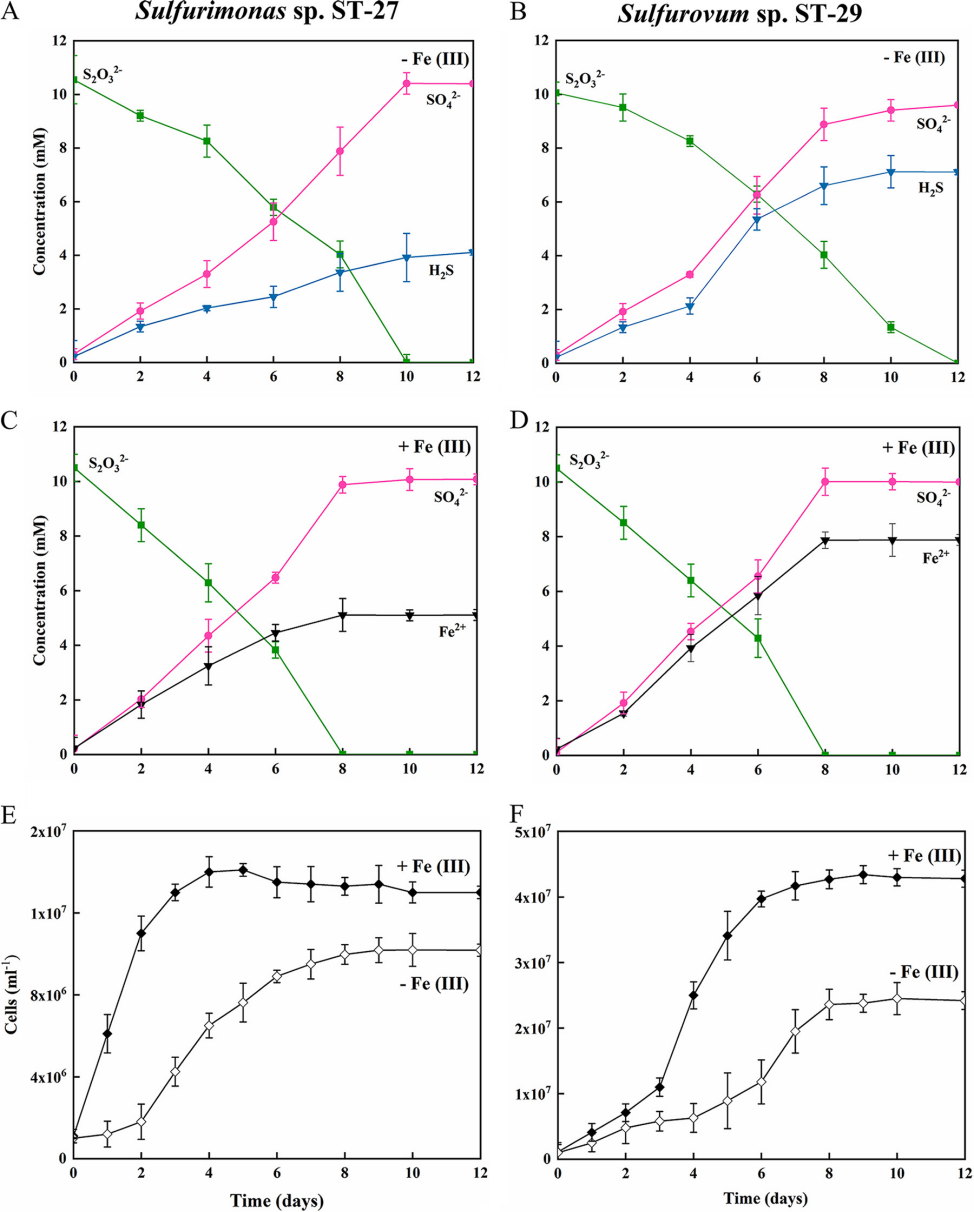
Thiosulfate disproportionation by strains ST-27 (left panel) and ST-29 (right panel). (A and B) Incubation without ferrihydrite. (C and D) Incubation with ferrihydrite. (E and F) Cell counts over time from three parallel incubations with and without ferrihydrite. The concentrations of thiosulfate (squares, green), sulfate (circles, red), dissolved sulfides (inverted triangles, blue), Fe (II) (inverted triangles, black) and cell counts (diamonds, solid and open) are shown over the course of the experiment. The data shown are the mean values for each condition, with error bars reflecting the standard errors of three biological replicates.

**FIG 4:**
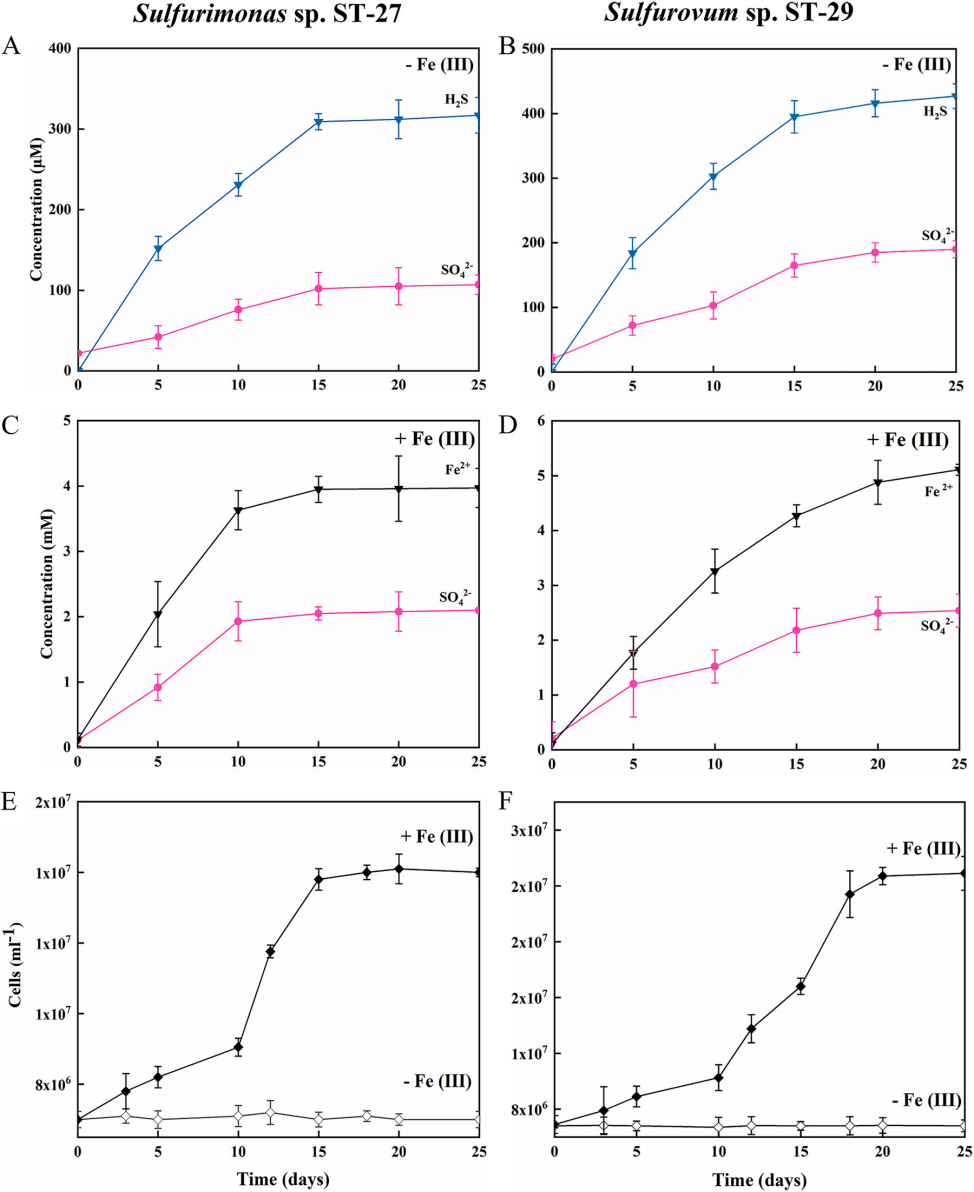
Elemental sulfur disproportionation by strains ST-27 (left panel) and ST-29 (right panel). (A and B) Incubation without ferrihydrite. (C and D) Incubation with ferrihydrite. (E and F) Cell counts over time from three parallel incubations performed with and without ferrihydrite. The concentrations of sulfate (circles, red), sulfides (inverted triangles, blue), Fe (II) (inverted triangles, black) and cell counts (diamonds, solid and hollow) are shown over the course of the experiment. The data shown are the mean values for each condition, with error bars reflecting the standard errors of three biological replicates.

To characterize the ability of the isolates to disproportionate sulfur, the five isolates described above were grown in the presence of different inorganic sulfur compounds as the sole energy source. The results showed that all strains could disproportionate thiosulfate and elemental sulfur but not sulfite (Table 1). Furthermore, 21 strains of different species of *Sulfurimonas* and *Sulfurovum*, including 9 type strains (publicly available) and 12 new isolates (this study), were also tested (Table 2). 12 of them were demonstrated to be able to disproportionate sulfur compounds. *Sulfurimonas* sp. ST-79 and *Sulfurimonas* sp. ST-367 from hydrothermal plumes, Sulfurovum riftiae 1812E^T^ from hydrothermal polychaete, and 7 isolates from mangrove sediments were found to grow vigorously by means of thiosulfate and elemental sulfur disproportionation (Table 2). *Sulfurimonas hydrogeniphila* NW10^T^ from a hydrothermal chimney sample and *Sulfurimonas* sp. B2 from a deep-sea sediment had relatively weak abilities for thiosulfate disproportionation, and both were unable to disproportionate S^0^ (Table 2). Taken together, these results show that the ability to disproportionate sulfur compounds may be common among members of the genera *Sulfurimonas* and *Sulfurovum*. We then selected the *Sulfurimonas* sp. ST-27 and *Sulfurovum* sp. ST-29 strains, as representatives of the predominant ASVs, to characterize the thiosulfate and elemental sulfur disproportionation processes (Fig. 3 and 4).

**TABLE 2:** Ability or inability to disproportionate different sulfur compounds (S_2_O_3_^2–^, SO_3_^2–^, and S^0^) in other species of the genera *Sulfurimonas* and *Sulfurovum*, including 9 type strains and 14 newly isolated strains in this study

Strains	Isolation site	Isolation environment	Strains with the highest 16S rRNA gene sequence similarity	Sulfur disproportionation^*a*^	No. in culture collections
Type strains					
*Sulfurimonas hydrogeniphila* NW10^T^	Northwestern Indian Ocean	Hydrothermal vent chimney		NaS_2_O_3_ (w)	MCCC 1A13987^T^ (KCTC 15781^T^)
*Sulfurimonas indica* NW8N^T^	Northwestern Indian Ocean	Hydrothermal vent chimney		–^*b*^	MCCC 1A13988^T^ (KCTC 15780^T^)
*Sulfurimonas sediminis* S2-6^T^	Southwestern Indian Ocean	Hydrothermal vent sediment		–	MCCC 1A14513^T^ (KCTC 15854^T^)
Sulfurimonas autotrophica OK10^T^	Mid-Okinawa Trough, Japan	Hydrothermal vent sediment		–	JCM 11897^T^ (DSM 16294^T^)
Sulfurimonas paralvinellae GO25^T^	Mid-Okinawa Trough, Japan	Hydrothermal vent polychaetes		–	JCM 13212^T^ (DSM 17229^T^)
*Sulfurimonas xiamenensis* 1-1N^1^	Xiamen island, China	Coastal sediment		–	MCCC 1A14514^T^ (KCTC 15851^T^)
*Sulfurimonas lithotrophica* GYSZ_1^T^	Xiamen island, China	Coastal sediment		–	MCCC 1A14739^T^ (KCTC 15853^T^)
*Sulfurovum indicum* ST-419^T^	Northwestern Indian Ocean	Hydrothermal vent plume		–	MCCC 1A17954^T^ (KCTC 25164^T^)
Sulfurovum riftiae 1812E^T^	East Pacific Rise	Hydrothermal vent polychaete		NaS_2_O_3_ (+) / S^0^ (+)	DSM 101780^T^ (JCM 30810^T^)
Newly isolated strains					
*Sulfurimona*s *marina* B2	South China Sea	Deep-sea sediment	*Sulfurimonas indica* NW8N^T^ (95.9%)	NaS_2_O_3_ (w)	MCCC 1A14515^T^ (KCTC 15852^T^)
*Sulfurimona*s sp. ST-49	Northwestern Indian Ocean	Hydrothermal vent plume	Sulfurimonas paralvinellae GO25^T^ (98.4%)	–	MCCC M28994
*Sulfurimona*s sp. ST-79	Northwestern Indian Ocean	Hydrothermal vent plume	Sulfurimonas paralvinellae GO25^T^ (97.1%)	NaS_2_O_3_ (+) / S^0^ (+)	MCCC M24543
*Sulfurimona*s sp. ST-367	Northwestern Indian Ocean	Hydrothermal vent plume	Sulfurimonas paralvinellae GO25^T^ (97.1%)	NaS_2_O_3_ (+) / S^0^ (+)	MCCC M23032
*Sulfurimona*s sp. SWIR-19	Southwestern Indian Ocean	Hydrothermal vent chimney	Sulfurimonas paralvinellae GO25^T^ (96.8%)	–	MCCC 1A16250
*Sulfurimona*s sp. HSL1-2	Zhangzhou island, China	Mangrove sediment	Sulfurimonas paralvinellae GO25^T^ (99.3%)	NaS_2_O_3_ (+) / S^0^ (+)	MCCC 1A19178
*Sulfurovum* sp. HSL1-3	Zhangzhou island, China	Mangrove sediment	Sulfurovum lithotrophicum ATCC BAA-797^T^ (94.9%)	NaS_2_O_3_ (++) / S^0^ (++)	MCCC M25234
*Sulfurimona*s sp. HSL1-6	Zhangzhou island, China	Mangrove sediment	*Sulfurimonas lithotrophica* GYSZ_1^T^ (93.9%)	NaS_2_O_3_ (++) / S^0^ (++)	MCCC 1A18693
*Sulfurimona*s sp. HSL1-7	Zhangzhou island, China	Mangrove sediment	Sulfurimonas paralvinellae GO25^T^ (95.5%)	NaS_2_O_3_ (+) / S^0^ (+)	MCCC 1A17955
*Sulfurimona*s sp. HSL3-1	Zhangzhou island, China	Mangrove sediment	*Sulfurimonas lithotrophica* GYSZ_1^T^ (93.6%)	NaS_2_O_3_ (+) / S^0^ (+)	MCCC 1A18844
*Sulfurimona*s sp. HSL3-2	Zhangzhou island, China	Mangrove sediment	*Sulfurimonas lithotrophica* GYSZ_1^T^ (95.4%)	NaS_2_O_3_ (+) / S^0^ (+)	MCCC 1A19147
*Sulfurimona*s sp. HSL3-7	Zhangzhou island, China	Mangrove sediment	Sulfurimonas paralvinellae GO25^T^ (92.7%)	NaS_2_O_3_ (++) / S^0^ (++)	MCCC 1A18694

a ++, strongly positive; +, positive; w, weakly positive; –, negative.

b dashes represented the cells growth is negative.

In the case of thiosulfate, both strains showed quite similar disproportionation rates in the presence and absence of ferrihydrite, and the growth efficiency was higher in the ferrihydrite-supplemented media than in the media without ferrihydrite. Without ferrihydrite, 10.61 mM thiosulfate was disproportionated to 10.43 mM sulfate and 4.10 mM dissolved sulfide by strain ST-27 over 12 days of incubation. Similarly, 9.64 mM sulfate and 7.12 mM dissolved sulfide were produced from 10.12 mM thiosulfate by strain ST-29 (Fig. 3A and B). The ratio of sulfate produced to thiosulfate consumed was approximately 1:1, which is in accordance with the theoretical stoichiometry of the reaction: S_2_O_3_^2–^ + H_2_O → SO_4_^2–^ + HS^–^ + H^+^. Since we only measured the dissolved sulfide here and did not measure the gaseous sulfide, it is normal that the measured sulfide production is lower than the predicted amount. The maximum cell densities reached 1.04 × 10^7^ cells mL^−1^ for strain ST-27 and 2.40 × 10^7^ cells mL^−1^ for strain ST-29 (Fig. 3E and F). Under these reactions, the doubling times of the two strains were 2.4 and 1.9 days, respectively. When ferrihydrite was added to the medium, sulfide accumulated as Fe-sulfide, and dissolved sulfide was not detectable (Fig. 3C and D). The maximal cell density was 1.36 × 10^7^ cells mL^−1^ for strain ST-27 and 4.28 × 10^7^ cells mL^−1^ for strain ST-29. Under these conditions, doubling times of strains ST-27 and ST-29 were 1.6 and 1.8 days, respectively (Fig. 3E and F). In the noninoculated controls, the initial thiosulfate concentration (10 mM) did not change, and no sulfide and sulfate were detected (data not shown).

With elemental sulfur, the disproportionation reaction occurred both in the presence and absence of ferrihydrite, but growth was only observed in the presence of ferrihydrite. Without ferrihydrite, 317 μM dissolved sulfide and 106 μM sulfate were produced by strain ST-27 over an incubation period of 25 days (Fig. 4A). Similarly, 426 μM dissolved sulfide and 189 μM sulfate were measured in the culture of strain ST-29 (Fig. 4B). When ferrihydrite was added, growth was observed, and the cell numbers reached a maximum of 1.41 × 10^7^ cells mL^−1^ for strain ST-27 and 2.48 × 10^7^ cells mL^−1^ for strain ST-29 over a 25-day incubation period (Fig. 4E and F). The growth was coupled with the production of approximately 3.97 mM Fe (II) and 2.10 mM sulfate by strain ST-27 and 5.11 mM Fe (II) and 2.54 mM sulfate by strain ST-29 (Fig. 4C and D), a ratio close to 1.9 to 2.0:1, as expected from the following reaction: 3S^0^ + 2 Fe(OH)_3_ → SO_4_^2–^ + 2FeS + 2H^+^ + 2H_2_O. Free sulfide was not detected during the experiment, suggesting that the produced sulfide was completely chelated, and the appearance of a greyish-black precipitate was observed. In the control treatments, no obvious increase of sulfate and sulfide products was observed (data not shown).

When isolates ST-27 and ST-29 were grown with thiosulfate or elemental sulfur as the sole energy source in the presence of ferrihydrite, the formation of black precipitates visible to the naked eye was observed. These black precipitates were not sensitive to magnetism and were further characterized by scanning electron microscopy (SEM), energy-dispersive spectrum (EDS) and X-ray diffraction (XRD), as detailed in Text S1 and in Fig. S3.

10.1128/msystems.00954-22.1 TEXT S1 Supplemental methods and results. Download TEXT S1, DOC file, 0.06 MB. Copyright © 2022 Wang et al. 2022 Wang et al. https://creativecommons.org/licenses/by/4.0/ This content is distributed under the terms of the Creative Commons Attribution 4.0 International license.

10.1128/msystems.00954-22.3 FIG S3 SEM images and XRD spectra of iron-Fe particulates after the growth of strains under thiosulfate (A–C, I) and elemental sulfur (D–F, J) disproportionation conditions. Panels A to F show the surface modifications of iron-sulfides under thiosulfate (A–C) and elemental sulfur (D–F) disproportionation conditions by SEM, respectively. Panels A and D, as the control (noninoculated), as well as Panels G and H showed the EDS spectra of the area indicated by the red square in panels B and E, respectively. The white arrow indicates cells. Each scale bar equals 10 μm in panels A to C, 30 μm in panels D to E, and 5.0 μm in panel F. In panel I, the red lines depict published mackinawite (FeS) reference peaks (44). The blue lines depict greigite (Fe_3_S_4_) reference peaks (64). The green lines depict marcasite (FeS_2_) reference peaks (65). In panel J, the red lines depict published pyrite (FeS_2_) reference peaks (66). The blue lines depict published iron sulfide (FeS) reference peaks (67). Download FIG S3, TIF file, 2.1 MB. Copyright © 2022 Wang et al. 2022 Wang et al. https://creativecommons.org/licenses/by/4.0/ This content is distributed under the terms of the Creative Commons Attribution 4.0 International license.

### Evaluation of the need for direct cell-S^0^ contact via dialysis tube experiments.

**FIG 5:**
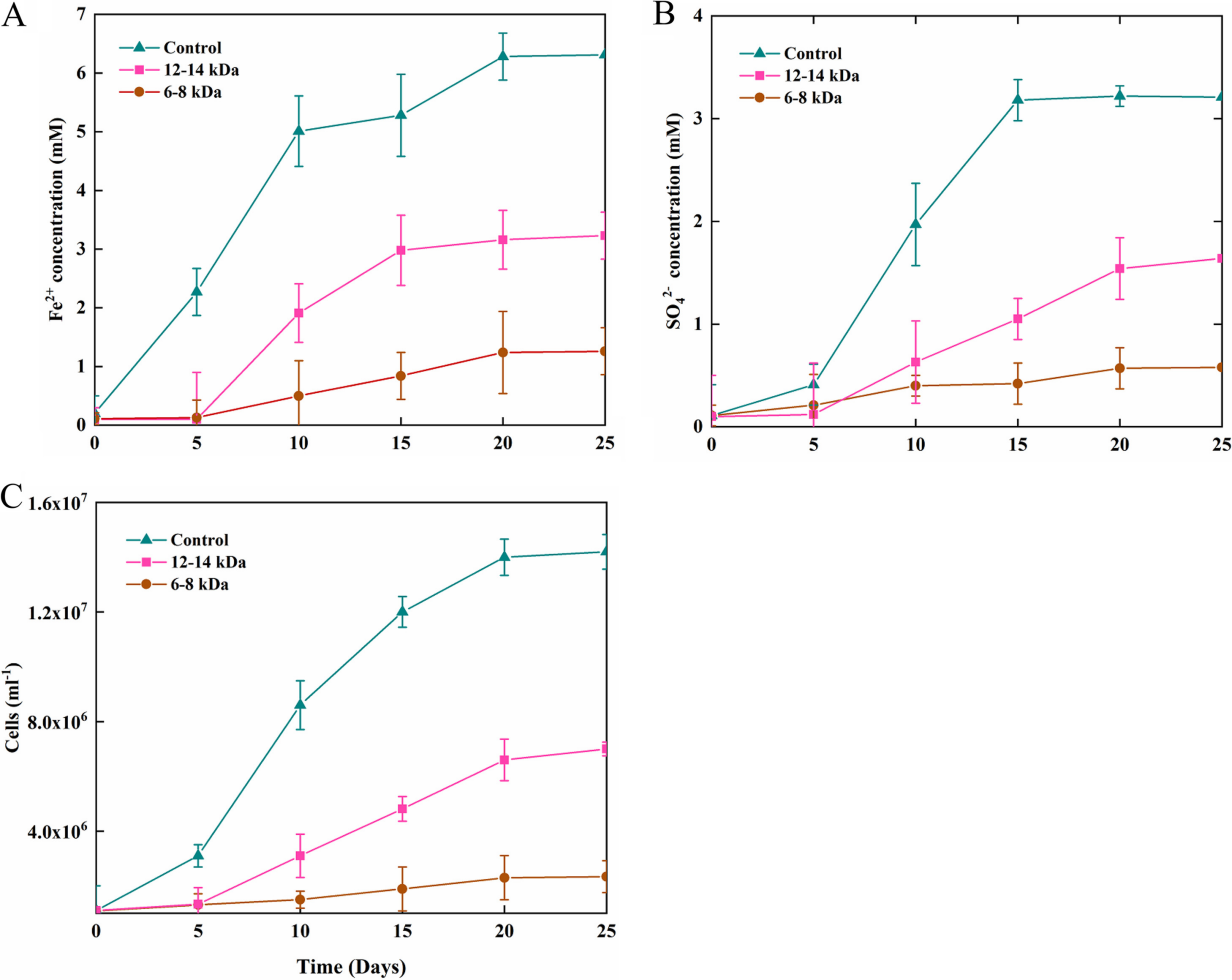
Fe (II) concentrations (A), sulfate concentrations, (B) and cell concentrations (C) in cultures of strain ST-27 grown with S^0^ provided as the sole electron donor and acceptor. S^0^ was either provided directly in the medium (control) or sequestered in dialysis membranes (pore sizes of 12 to 14 kDa or 6 to 8 kDa) to prevent physical contact with cells.

An experiment was performed with strain ST-27 to determine whether a direct contact to S^0^ was required for growth when S^0^ was supplied (i) as an electron donor (sulfur oxidation), (ii) as an electron acceptor (sulfur reduction), or (iii) as both an electron donor and an electron acceptor (S^0^ disproportionation). The results demonstrated that cell growth via S^0^ disproportionation did not require direct physical contact with sulfur particles (Fig. 5A–C). However, the efficiency of sulfur disproportionation decreased when the cells were separated from S^0^, and this was indicated by less ferrous iron being released, lower sulfate production, and a lower cell density, compared to positive growth controls (without a dialysis membrane). The smaller the pore size of the dialysis membrane, the greater the decrease in the disproportionation reaction was observed. In detail, the ferrous iron production decreased by 51% and 20% when S^0^ was sequestered in dialysis tubing with pore sizes of 6 to 8 kDa and 12 to 14 kDa, respectively (Fig. 5A). Correspondingly, the sulfate concentration decreased by 50% and 18%, respectively (Fig. 5B), and the cell densities decreased by 49% and 16%, respectively (Fig. 5C). Similarly, the direct contact was also unessential in S^0^ reduction when H_2_ was used as the electron donor (Fig. S4). Nevertheless, under these conditions, cell growth was also inversely correlated with the pore size in cases involving higher sulfur reduction rates with larger pores (Fig. S4). In contrast, no cell growth was observed when S^0^ was sequestered in dialysis tubing and S^0^ was used as the electron donor with 4% O_2_ as the electron acceptor (Fig. S5). Thus, the disproportionation and reduction of S^0^ do not strictly require direct contact with the mineral but do appear to be promoted by it, whereas the oxidation of S^0^ absolutely requires direct contact with the mineral. Similar results were obtained with strain ST-29 (data not shown).

10.1128/msystems.00954-22.4 FIG S4 Sulfide concentrations (A) and cell densities (B) in cultures of strain ST-27 grown autotrophically with H_2_ as the electron donor and S^0^ as the electron acceptor. S^0^ was either provided directly in the medium (control) or sequestered in dialysis membranes to prevent physical contact with cells. Download FIG S4, TIF file, 2.8 MB. Copyright © 2022 Wang et al. 2022 Wang et al. https://creativecommons.org/licenses/by/4.0/ This content is distributed under the terms of the Creative Commons Attribution 4.0 International license.

10.1128/msystems.00954-22.5 FIG S5 Sulfate concentrations (A) and cell numbers (B) in cultures of strain ST-27 cultivated autotrophically with S^0^ as the electron donor and 4% O_2_ as the electron acceptor. S^0^ was either provided directly in the medium (control) or sequestered in dialysis membranes to prevent physical contact with cells. Download FIG S5, TIF file, 2.7 MB. Copyright © 2022 Wang et al. 2022 Wang et al. https://creativecommons.org/licenses/by/4.0/ This content is distributed under the terms of the Creative Commons Attribution 4.0 International license.

### A comparative genomic analysis of our isolates and the confirmed and putative SDB of the phyla *Desulfobacterota*, *Firmicutes*, and *Campylobacterota*.

**FIG 6:**
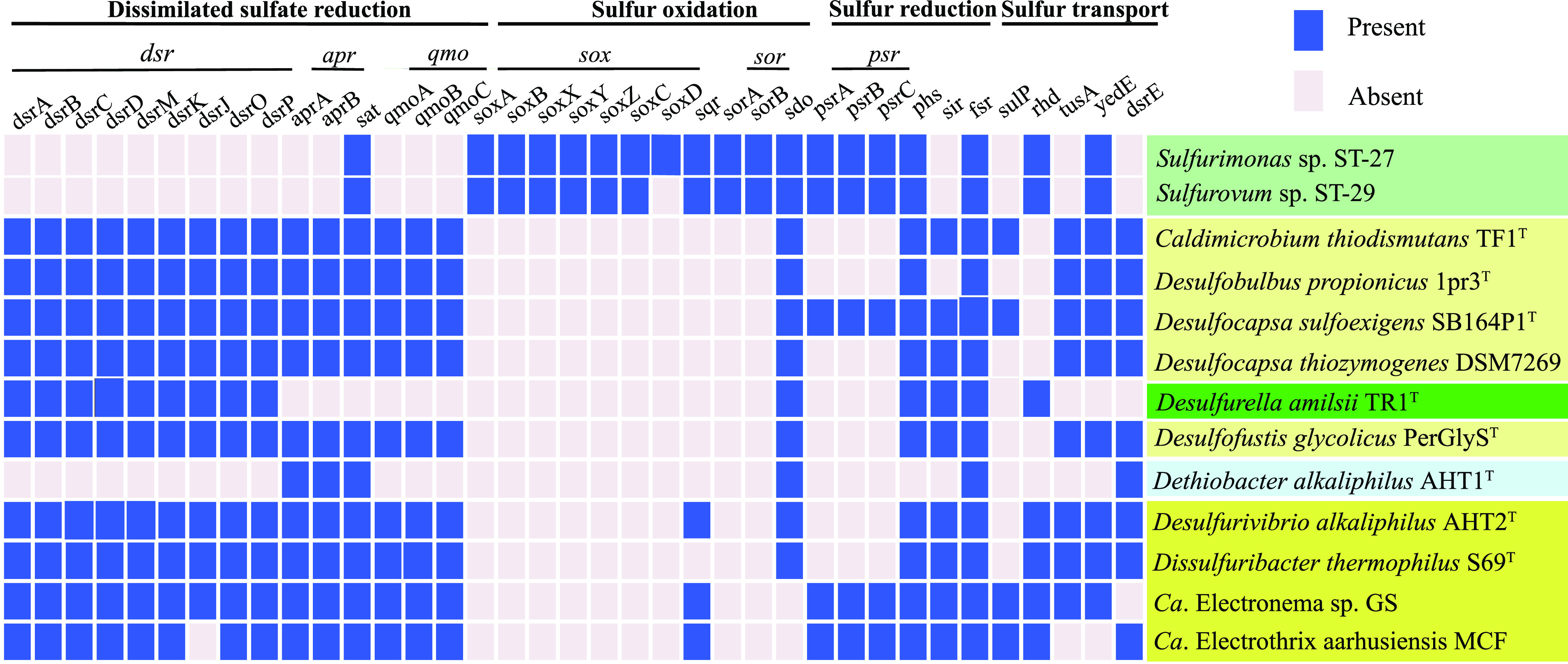
Heat map showing the presence (blue) or absence (gray) of protein-encoding genes involved in sulfur metabolism in the ST-27 and ST-29 strains isolated in this study as well as in 11 confirmed or putative S^0^-disproportionating species of the phyla *Desulfobacterota*, *Firmicutes*, and *Campylobacterota*. The campylobacterotal strains isolated in this study and in a previous study are shown on light and dark green backgrounds, respectively. S^0^-disproportionators belonging to *Firmicutes* are represented on a blue background. Putative or confirmed S^0^-disproportionators belonging to *Desulfobacterota* are shown on light yellow and dark yellow backgrounds. Strains with a dark yellow background also share the ability to perform sulfur oxidation.

Genomic analyses showed that strains ST-27 and ST-29 contained genes that are essential for the oxidation of reduced sulfur compounds, such as the genes *soxABCDXYZ*, *sorAB*, *sqr*, and *sdo* (Fig. 6). The homologs of the genes *psrABC*, *phsAB*, and *fsr*, which are, respectively, responsible for the reduction of polysulfide, the disproportionation of thiosulfate, and the reduction of sulfite, were also present in two genomes (Fig. 6). In addition, both strains contained the genes *yedE* and *rhd*, which are involved in sulfur transfer in some bacterial models (Fig. 6). However, the two strains lacked some genes of the dissimilatory sulfate reduction pathway, including *aprAB*, *dsrABD*, *dsrС*, and *dsrMKJOP*, which are currently regarded as involved in the sulfur disproportionation pathway in some bacterial strains (11). Furthermore, a comparative genomic analysis showed a main difference in gene content between the two strains isolated in this work and the 11 S^0^-disproportionating bacteria belonging to the phyla *Desulfobacterota* (9), *Firmicutes* (1), and *Campylobacterota* (1) (Fig. 6). Almost all of the S^0^-disproportionating bacteria analyzed here contain a complete dissimilatory sulfate reduction pathway, with the exceptions of Desulfurella amilsii (*Campylobacterota*) and Dethiobacter alkaliphilus (*Firmicutes*), whereas genes encoding sulfur oxidation, such as the *sox* gene cluster and the *sor* gene, are totally absent in the genomes of representatives of the phylum *Desulfobacterota* (Fig. 6). The *sqr* gene is encoded in the genomes of Desulfurivibrio alkaliphilus and two putative sulfur disproportionators, namely, *Candidatus* Electronema sp. GS and *Candidatus* Electrothrix aarhusiensis, and it gives them the ability to oxidize sulfur (Fig. 6). The sulfur reduction genes *phsA* and *psrA* were encoded in all of the genomes analyzed, with the exception of Dethiobacter alkaliphilus. In addition, most of the microorganisms share genes that code for proteins that are involved in the activation of insoluble sulfur compounds and in the transport of such compounds (Fig. 6). Thus, based on the above analysis, *Campylobacterota* strains ST-27 and ST-29 may possess an unknown sulfur disproportionation pathway that may be quite different from that which has already been partially described in other sulfur disproportionators.

## DISCUSSION

In this study, we demonstrated that sulfur disproportionation can be carried out by members of the phylum *Campylobacterota*, which are ubiquitous and well-known as the major chemolithoautotrophic sulfur oxidizers in hydrothermal vent environments (32, 33). Prior to this study, the disproportionation of inorganic sulfur compounds appeared to be a fairly common metabolic trait in sulfate-reducers affiliated with the *Desulfobacterota.* Only a few sulfur-oxidizing taxa that performed sulfur-dependent dissimilatory nitrate reduction to ammonium (or DNRA), namely, Desulfurivibrio alkaliphilus and Dissulfuribacter thermophilus, were found to disproportionate sulfur compounds (34, 35). It has also been suggested that the cable bacterial cells “*Candidatus* Electronema” and “*Candidatus* Electrothrix” could also hypothetically disproportionate sulfur to conserve energy when they cannot perform sulfide oxidation (36). Herein, the capacity of *Campylobacterota* isolates to grow chemolithoautotrophically via sulfur disproportionation was demonstrated in addition to the capacities of sulfur oxidation and sulfur reduction. These capacities give these taxa a metabolic versatility that allows them to adapt to changing environmental conditions in the dynamic hydrothermal vent habitat. Thus, our results extend our knowledge on the diversity of sulfur disproportionators and demonstrate the metabolic versatility of some hydrothermal chemolithoautotrophs by showing that they can intervene at different levels of the microbial sulfur cycle; indeed, some microorganisms, such as strains ST-27 and ST-29, are capable of oxidizing, disproportionating, and reducing inorganic sulfur compounds.

Culture-dependent and culture-independent studies showed that the mesophilic chemolithoautotrophic genera *Sulfurimonas* and *Sulfurovum* are widespread and predominant sulfur-oxidizing bacteria in the global deep-sea hydrothermal ecosystem (37). They are especially abundant in vent plumes and are often associated as symbionts with animals. For example, the genera *Sulfurimonas* and *Sulfurovum* accounted for 20% and 28% of the relative bacterial abundance, respectively, in diffuse fluids of the Axial Seamount on the Juan de Fuca Ridge (38). In addition, *Sulfurimonas* and *Sulfurovum* accounted for up to 61.60% and 19.76% of the total bacterial abundance in the Carlsberg Ridge vent plumes in the northwest Indian Ocean (39). In this study, it is intriguing that they were the most dominant members in all of the sulfur disproportionating enrichment cultures from the vent plumes (Fig. 1). Additionally, we conducted research on the Integrated Microbial Next Generation Sequencing (IMNGS) platform (https://www.imngs.org/) and found that strain ST-27-like sequences and strain ST-29-like sequences (>97% similarity of 16S rRNA genes) were ubiquitous in a variety of environments, including marine sediments, deep-sea hydrothermal vents, freshwaters, and activated sludge. We also found that these were dominant (>1% abundance) in marine sediments and in deep-sea hydrothermal vents. Therefore, it is possible that microbial sulfur disproportionation by chemolithoautotrophic *Sulfurimonas* and *Sulfurovum* bacteria is widespread in anaerobic environments and plays a significant role in deep-sea hydrothermal vents and in some marine sediments. In addition, 21 strains of different species of *Sulfurimonas* and *Sulfurovum*, including 9 type strains (publicly available) and 12 new isolates (this study) from hydrothermal vents or other natural environments, were also tested, and 12 of them were demonstrated to be able to disproportionate sulfur compounds (Table 2). These results tend to support the hypotheses that the disproportionation of inorganic sulfur compounds could be more widespread in anaerobic environments than is known and that the chemolithoautotrophic genera *Sulfurimonas* and *Sulfurovum* might play a role in this process.

The biochemical pathways of sulfur disproportionation are partially described in some taxa (11), but the mechanism involved in the initial step of S^0^ disproportionation remains unknown in most microorganisms. Considering the extremely low solubility and reactivity of S^0^ (1 μg L^−1^ at 25°C and 15 μg L^−1^ at 80°C), microorganisms most likely require a specific activation mechanism to make S^0^ available for their energy metabolism (40). Four mechanisms have been postulated to cope with the low solubility of elemental sulfur for its reduction (13), and these could also be extended to its disproportionation: (i) a direct physical attachment and interaction of the cells with the solid S^0^ phase to generate soluble polysulfanes; (ii) a direct uptake of polymeric sulfur; (iii) a soluble intermediate form of sulfur, such as polysulfide, that is generated via the nucleophilic attack of S^0^ by sulfide; and (iv) an extracellular electron transport from the sulfur via a pilus. Here, the results indicate that S^0^ disproportionation can be conducted with direct or indirect contact with S^0^. In addition, direct contact with sulfur seems beneficial to the reaction, as maximum efficiency is obtained under this condition, whereas the efficiency of S^0^ disproportionation decreases as the membrane pore size decreases. These results do not allow for the determination of the nature of the real substrate used for the S^0^ disproportionation reaction (i.e., whether the S^0^ is activated and used directly or transformed into a more soluble molecule before entering the cell). If elemental sulfur is indeed the direct substrate of the reaction (in which case it would enter directly into the cell via a transporter or would release electrons) (41), the decrease in the efficiency of the reaction in the dialysis membrane could be due to the fact that only the sulfur nanoparticles that could pass through the pores could be used. Alternatively, if S^0^ enters the cell after conversion to other, more soluble linear molecules (e.g., polysulfides, polysulfanes), the decrease in reaction efficiency could be explained in that the generation of these more soluble forms of sulfur would occur in the immediate vicinity of the cells from the sulfur nanoparticles that have passed through the membrane. In conclusion, the activation mechanism of elemental sulfur requires further investigation.

To date, some key enzymes involved in the thiosulfate and sulfite disproportionation pathways have been revealed by biochemical studies on a small number of strains (10). However, the enzymatic machinery of elemental sulfur disproportionation has been much less studied from a functional perspective. Based on the current state of knowledge, there are clearly several routes of disproportionation for inorganic sulfur compounds, as are reviewed elsewhere (10, 11, 23, 42). The bacterial sulfur disproportionators originating from hydrothermal vents that have been described so far (see the list in the Introduction section) and belong to *Desulfobacterota* most likely use some enzymes that are common to the dissimilatory sulfate reduction pathway (*aprAB*, *sat*, *dsrABD*, *dsrС*, and *dsrMKJOP*) to carry out parts of the reactions of the sulfur disproportionation pathway (11, 23, 42). Other candidate genes (rhodanese-like sulfurtransferases, molybdopterin oxidoreductases, YTD gene cluster, MOLY cluster, *aprB* gene with a truncated tail, and Eyh protein) (11–13, 23, 42) that could encode proteins that are putatively involved in the sulfur disproportionation pathway have also been reported. The genomes of the strains ST-27 and ST-29 that were isolated in this study, which belong to *Campylobacterota*, do not encode the *aprAB*, *dsrAB*, *dsrС*, and *dsrMKJOP* genes (Fig. 6). Instead, they include genes that encode proteins that are involved in sulfur oxidation (such as the *sox* gene cluster, *sorAB*, and *sdo*), sulfur reduction (*psrABC*, *phsAB*, and *fsr*), and the sulfur relay (*yedE* and *rhd*) as well as unidentified genes that may participate in the process of sulfur disproportionation in these taxa. Functional investigations will be required in order to draw conclusions. These results suggest the existence of an unrevealed catabolic pathway for sulfur disproportionation.

**FIG 7:**
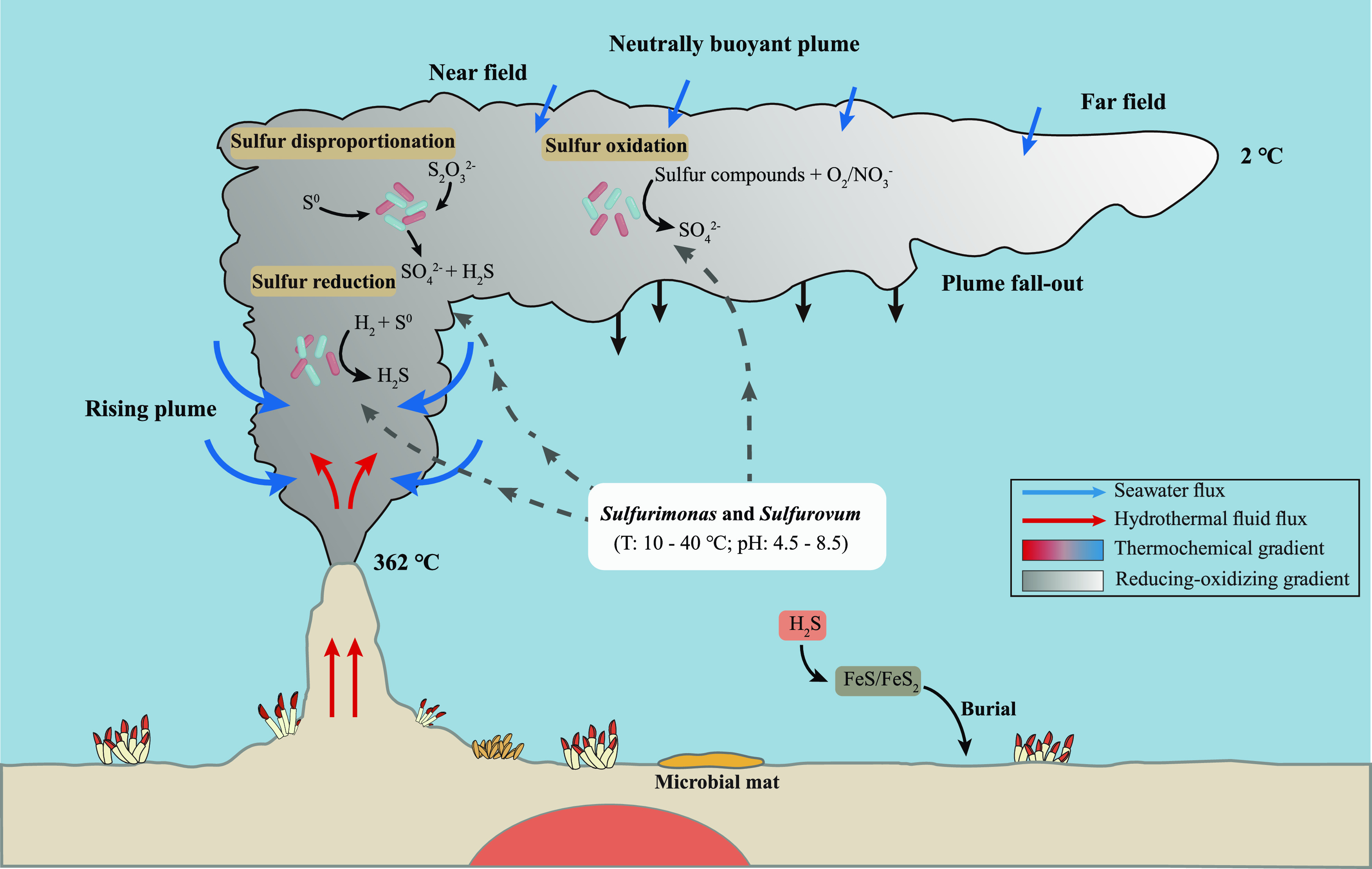
Possible ecological niches for the genera *Sulfurimonas* and *Sulfurovum* to achieve the oxidation, reduction, and disproportionation of sulfur in the deep-sea hydrothermal plume.

Hydrothermal plumes undergo rapid cooling and geochemical changes during their turbulent mixing of hydrothermal fluids and seawater, making them a dynamic habitat (43). Under the action of thermal buoyancy, the high-temperature fluid rises for hundreds of meters until it reaches buoyancy balance with the surrounding environment, and it then spreads thousands of kilometers along isopycnals (44). Even after plume buoyancy ends in the near field, the oxidation processes continue (Fig. 7), and in the far field, all of the reduced components are oxidized, and background oceanic processes override the hydrothermal processes (Fig. 7). Thus, there are a series of environmental gradients of temperature, redox potential, sulfur species, and ions in both the rising plume and the neutrally buoyant plume (Fig. 7). Furthermore, inorganic sulfur compounds, including elemental sulfur and thiosulfate, are present (45), and the efficient sulfide-scavenging manganese (IV) and iron (III) minerals are abundant (46). Thus, it is likely that the process of sulfur disproportionation occurs in the plume ecosystem. *Sulfurimonas* and *Sulfurovum*, as dominant members in vent plumes, are involved in sulfur oxidation, sulfur reduction, and sulfur disproportionation, as revealed in this study. Thus, they may occupy different ecological niches and may participate in different processes of the sulfur cycle in hydrothermal vent ecosystems. We hypothesize that an ecological niche exists for sulfur disproportionation by *Sulfurimonas* and *Sulfurovum*, which would be one in which the availability of usable electron donors (H_2_) or acceptors (O_2_ or NO_3_^–^) is limited and only sulfur compounds that are in an intermediary oxidation state are abundant (Fig. 7). Furthermore, sulfur disproportionation could be an accessory metabolism for *Campylobacterota* bacteria that allows them to wait for more favorable conditions, as the process of sulfur disproportionation is much less energetically favorable than are the processes of sulfur oxidation and sulfur reduction.

In summary, some species of chemolithoautotrophic *Campylobacterota*, which are ubiquitous and abundant members of deep-sea marine hydrothermal ecosystems, can grow via sulfur disproportionation and can support primary production in vent ecosystems through this catabolism in addition to others that have already been documented. The ability to produce energy from only thiosulfate or elemental sulfur as the sole electron donor and acceptor by *Sulfurimonas* sp. and *Sulfurovum* sp. probably facilitates their adaptation to a wide range of habitats in which sulfur compounds are abundant and other electron donors and acceptors are rare. This study expands our knowledge on the global biogeochemical cycle of sulfur and provides new information on the catabolism and ecological places of major players in the hydrothermal ecosystem. In the future, omics analyses that are based on the sulfur disproportionators that were isolated here will be performed in order to explore their catabolic pathways.

## MATERIALS AND METHODS

### Sample collection and enrichment cultures.

9 hydrothermal plume samples were collected on September 11 and 26 of 2018, during the Chinese COMRA cruise DY57-Leg II by the research vessel *Zhukezhen* from the active Wocan hydrothermal field on the Carlsberg Ridge in the northwest Indian Ocean. Sampling information with specific descriptions is provided in Table S2. After being collected, the plume samples were placed in an insulated box on the submersible’s basket. Then, the water samples were immediately transferred into MMJS liquid medium, which was supplemented with elemental sulfur (Sigma-Aldrich, 1% wt/vol) as the sole substrate and poorly crystalline iron (III) oxide (ferrihydrite, 20 mM) as a sulfide trap under the gas phase of 80% N_2_/20% CO_2_ (200 kPa). The composition of the MMJ basal medium and the preparation technique are described in detail in Text S1. The ferrihydrite was prepared by neutralizing an FeCl_3_ solution with NaOH, and this was followed by desalination with water (47). No reducing agents were added to the medium, but all of the medium components, with the exceptions of NaHCO_3_, trace elements, and vitamins, were boiled for less than 5 min under a gas stream of 100% N_2_ in order to remove dissolved oxygen. The pH was adjusted to between 7.0 and 7.3. After the enrichment cultures were cultivated at 28°C for 7 to 14 days, the color of the ferrihydrite changed from brown to black, indicating Fe (III) reduction. The elemental sulfur also shifted from a yellow color to green. 2% (vol/vol) of each enrichment culture was then transferred into the same fresh medium and incubated at 28°C. A second subculture was carried out under exactly the same conditions when growth was observed in the first subculture after the initial enrichment.

10.1128/msystems.00954-22.7 TABLE S2 Detailed information on the sampling sites. Download Table S2, DOCX file, 0.01 MB. Copyright © 2022 Wang et al. 2022 Wang et al. https://creativecommons.org/licenses/by/4.0/ This content is distributed under the terms of the Creative Commons Attribution 4.0 International license.

During the third round of subculture in the laboratory, the cell survival status was estimated with a LIVE/DEAD BacLight Bacterial Viability Kit (Thermo Scientific, USA) (48). Cultures were added to the dye solution for LIVE/DEAD cells, following the manufacturer’s instructions, stained for 20 min in the dark, and then observed under a fluorescence microscope (Leica, Germany) (magnification: 40×; filter: 525 nm). The sulfate concentration was measured via ion chromatography (as described below) in all of the cultures in which the ferrihydrite turned black.

### DNA extraction and high-throughput sequencing analyses.

The bacterial community compositions of the stabilized enrichment cultures at the end of the third incubations were analyzed as follows. Genomic DNA was extracted using a MoBio PowerSoil DNA Extraction Kit (Qiagen, Germany), following the manufacturer’s instructions. The V3-V4 region of the bacterial 16S rRNA gene was amplified with the universal primers 338F (5′-ACTCCTRCGGGAGGCAGCAG-3′) and 806R (5′-GGACTACCVGGGTATCTAAT-3′) (49). The amplification of the 16S rRNA gene was performed as follows: initial denaturation at 95°C for 3 min; 27 cycles of denaturing at 95°C for 30 s, extension at 72°C for 45 s, and single extension at 72°C for 10 min; ending at 4°C. The polymerase chain reaction (PCR) products were purified using an AxyPrepDNA Gel Extraction Kit (Axygen Biosciences, USA), according to the instructions. Purified amplicons were sequenced on an Illumina MiSeq platform using 2 × 300 bp chemistry. The sequencing outputs were the raw sequence data.

After demultiplexing, the resulting sequences were quality filtered using fastp (0.19.6) (50) and were merged using FLASH (v1.2.11) (51). The high-quality sequences were then denoised using the DADA2 (52) plugin in the Qiime 2 (v. 2020.2) (53) pipeline with the recommended parameters, and this provided single nucleotide resolution, based on the error profiles within samples. The DADA2 denoised sequences are usually called amplicon sequence variants (ASVs). The taxonomic assignment of the ASVs was performed using the naive Bayes consensus taxonomy classifier that is implemented in Qiime2 and the SILVA 16S rRNA database (v138). The relative abundance of individual taxa within each community was estimated by comparing the number of sequences assigned to a specific taxon and the number of total sequences obtained for that sample. Alpha diversity indices, including the Chao1 and Shannon indices, were calculated to estimate the diversity and the richness of the bacterial communities in the different cultures. The data analysis process of Qiime 2 was performed using the Majorbio Cloud Platform (www.majorbio.com).

### Isolation of sulfur-disproportionating bacteria and taxonomic analysis.

For the bacterial isolation, 1 mL of the culture of each consortium was sampled from the third incubation cultures, transferred into 10 mL of MMJS liquid medium with elemental sulfur and ferrihydrite as described above, and incubated at 28°C. The well-grown culture was purified using the dilution-to-extinction technique with the same medium (54). The purity of the isolates was confirmed via microscopic examination and 16S rRNA gene sequencing.

The genomic DNA from the isolates was prepared using a DNA Extraction Kit (SBS Genetech, China), following the manufacturer’s instructions, and the 16S rRNA gene amplification was performed via PCR using the primer pair 27F (5′-AGAGTTTGATCCTGGCTCAG-3′) and 1492R (5′-TACGGCTACCTTGTTACGACT-3′). The PCR conditions (30 cycles of 45 s at 94°C, 45 s at 55°C, and 90 s at 72°C) were performed using a thermal cycler. The PCR products were purified and sequenced (55). To clarify the evolutionary relationships among the dominant species according to the ASVs and the isolates, a phylogenetic tree based on the 16S rRNA gene sequences was constructed via the neighbor-joining (NJ) method and the Kimura 2-parameter model, using MEGA 6.0 with the bootstrap values being based on 1,000 replicates (56).

### Sulfur disproportionation activity assays on isolates.

The abilities of isolates to grow via the disproportionation of thiosulfate (10 mM), elemental sulfur (1% wt/vol), and sulfite (1, 5 mM) were tested with the MMJS medium in the absence or presence of Fe (III). All of the experiments were performed in duplicate. In addition, appropriate control experiments were performed in order to differentiate biotic and abiotic reactions. Serum bottles were homogenized via manual shaking, and aliquots of the cultures were sampled using a syringe that was nitrogen-flushed prior to sampling. The samples were processed immediately for further analyses in order to minimize oxygen exposure. Bacterial growth was measured via direct cell counts using a phase-contrast microscope (Eclipse 80i, Nikon). Before counting, the iron precipitates were dissolved using a dithionite solution (5% in 0.3 M acetic acid) (57). The dissolved sulfide concentration was determined spectrophotometrically using the methylene blue method, according to Cline (1969) (58). Thiosulfate and sulfate were analyzed via ion chromatography (Dionex, USA) after separation on an IonPac AG19 column (4 × 250 mm) with NaOH (20 mM) as the eluent at a flow rate of 1 mL/min. Fe (III) reduction was monitored by quantitatively measuring Fe (II) using the ferrozine method (59). The samples for the assay were prepared as described previously (60).

### Morphological and chemical characterization of iron-sulfides particles.

The iron-sulfides particles formed in the sulfur disproportionation cultures were characterized via scanning electron microscopy (SEM, Hitachi, Japan), an energy-dispersive spectrum (EDS, IXRF Systems, USA), and X-ray diffraction (XRD, Physical Electronics, USA). The samples for the assay were prepared as described in Text S1.

### Experiment with dialysis membranes to test cell contact with S^0^.

Elemental sulfur was enclosed in dialysis tubing in batch cultures to examine the need for physical contact between the strains and the bulk solid sulfur. Three different growth conditions were examined as described above. S^0^ was added to dialysis membranes (Spectrum Laboratories, USA) with pore sizes of 6 to 8 kDa and 12 to 14 kDa, and this was followed by closure with dialysis clips. Before use, all of the dialysis membranes were incubated at 80°C in sterile deionized water for 24 h to remove preservatives, and this process was repeated three times, with the deionized water being replaced each time (61). Cultures grown with elemental sulfur and exposed fully to the medium (no dialysis membrane) were used as positive controls. Sterile, uninoculated media containing sulfur that was sequestered in dialysis membranes were used as negative controls. All of the treatments were performed in triplicate. The integrity of the dialysis membranes was checked after each experiment via visual inspection and SEM observation. The sulfide, ferrous iron, and sulfate production, as well as the cell density, were monitored as described above.

### Comparative genomics analyses.

The gene annotation was performed using the Rapid Annotation using Subsystem Technology (RAST) server (62) and the NCBI Prokaryotic Genomes Automatic Annotation Pipeline (PGAAP). The predicted CDSs were compared to the UniProtKB database, and functional predictions were then made using the InterProScan webserver (https://www.ebi.ac.uk/interpro/). Genes encoding proteins that are involved in dissimilatory sulfate reduction, sulfur oxidation, sulfur reduction, and sulfur transport were identified via a local BLASTp protein similarity search, using the reference sequences obtained from NCBI as queries. For the BLASTp analysis, we used an amino acid similarity cutoff of >20%, an alignment coverage of >80%, and an E value cutoff of 1E–5. Additionally, the obtained putative homologs from the genome were also compared against the NCBI database in order to obtain information on the protein domains that were present. This information was used as an extra confirmation of their functional gene identities. Then, the presence and absence of all of these functional genes, tested in all of the genomes, were represented as a heat map that was generated using TBtools (63).

Comparative genomics were carried out with genomes of taxa from various phyla. In this paper, the classification of these taxa is that of GTDB v. 207 (https://gtdb.ecogenomic.org/). The annotated reference genomes of the (i) *Desulfobacterota* strains, namely, Caldimicrobium thiodismutans TF1^T^ (AP014945.1), Desulfobulbus propionicus 1pr3^T^ (CP002364.1), *Desulfocapsa sulfoexigens* SB164P1^T^ (CP003985.1), Desulfofustis glycolicus PerGlyS^T^ (FQXS00000000.1), Desulfurivibrio alkaliphilus AHT2^T^ (CP001940.1), Dissulfuribacter thermophilus S69^T^ (MAGO00000000.1), *Candidatus* Electronema sp. GS (NQJD00000000.1), and *Candidatus* Electrothrix aarhusiensis MCF (MTKO00000000.1); (ii) *Campylobacterota* strain Desulfurella amilsii TR1^T^ (MDSU00000000.1); and (iii) *Firmicutes* strain Dethiobacter alkaliphilus AHT1^T^ (ACJM00000000.1), were retrieved from GenBank. The genome of Desulfocapsa thiozymogenes DSM 7269 (2514885009) was downloaded from IMG.

### Data availability.

All of the isolates were stored with 20% glycerol (vol/vol) at −80°C and were deposited in the Marine Culture Collection of China (MCCC) under collection numbers MCCC M25443, MCCC M25444, MCCC M27207, MCCC M27208 and MCCC M27209.

All of the Illumina sequence data from this study were deposited in the NCBI Sequence Read Archive (SRA) under accession numbers SRR19399983 to SRR19399988. The Sanger sequences of the genes encoding the 16S rRNA of the isolates were deposited to GenBank under the accession numbers ON631962, ON631964, ON632004, ON632007, and ON632008.
