# Supplementary figures and images for: Disproportionation of Inorganic Sulfur Compounds by Mesophilic Chemolithoautotrophic Campylobacterota
Source: mSystems. 2022 Dec 21;8(1):e00954-22. doi: 10.1128/msystems.00954-22 (PMC9948710; doi:10.1128/msystems.00954-22)

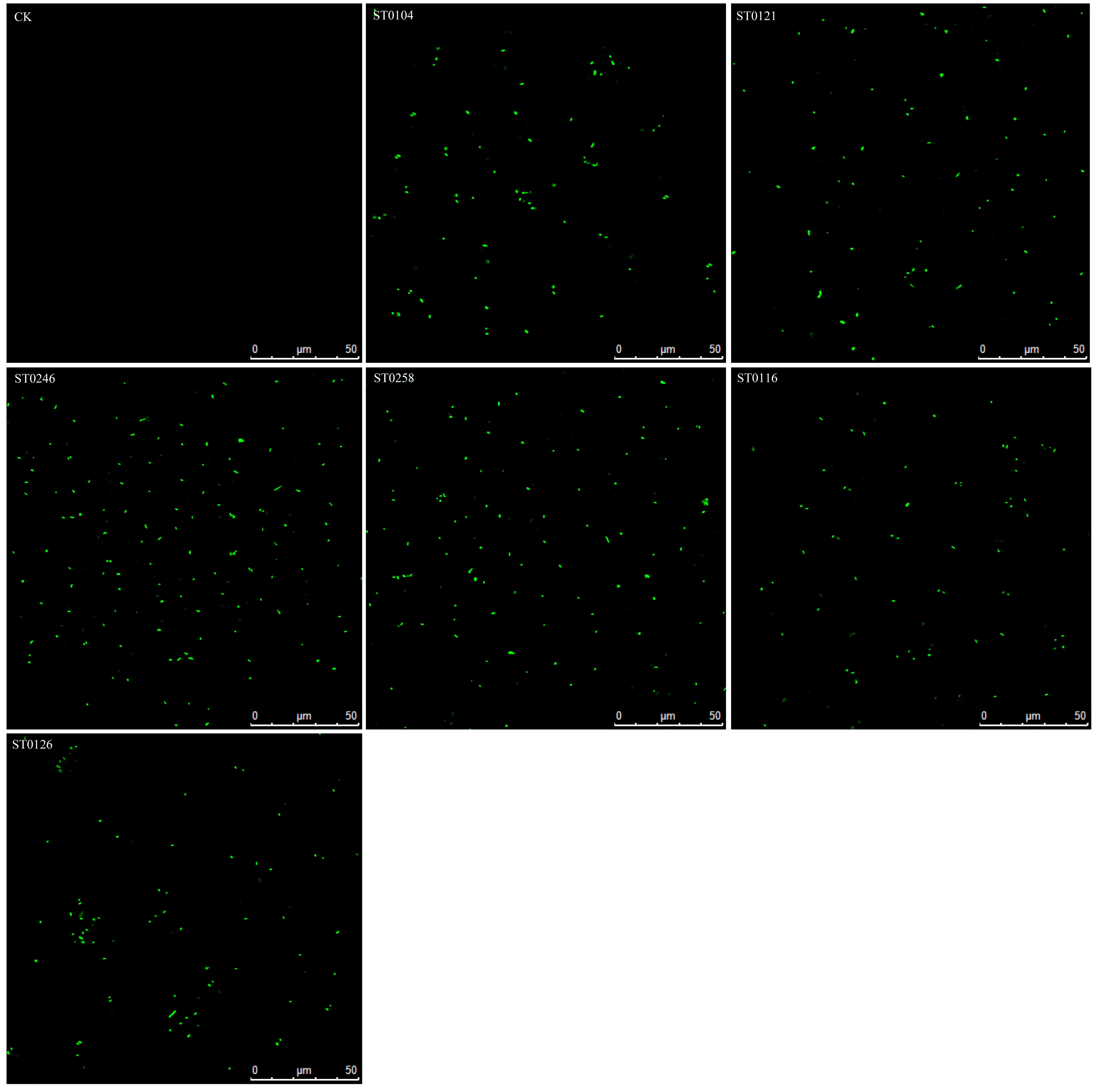

Supplement: FIG S2 [file msystems.00954-22-s0002.tif]

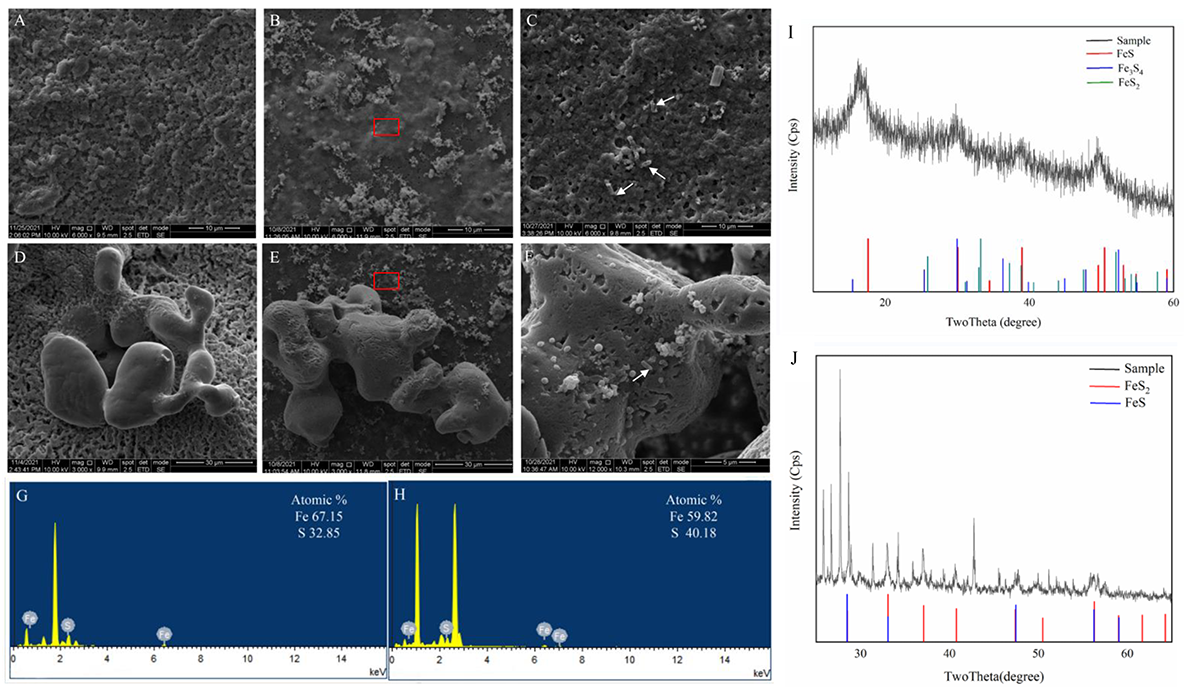

Supplement: FIG S3 [file msystems.00954-22-s0003.tif]

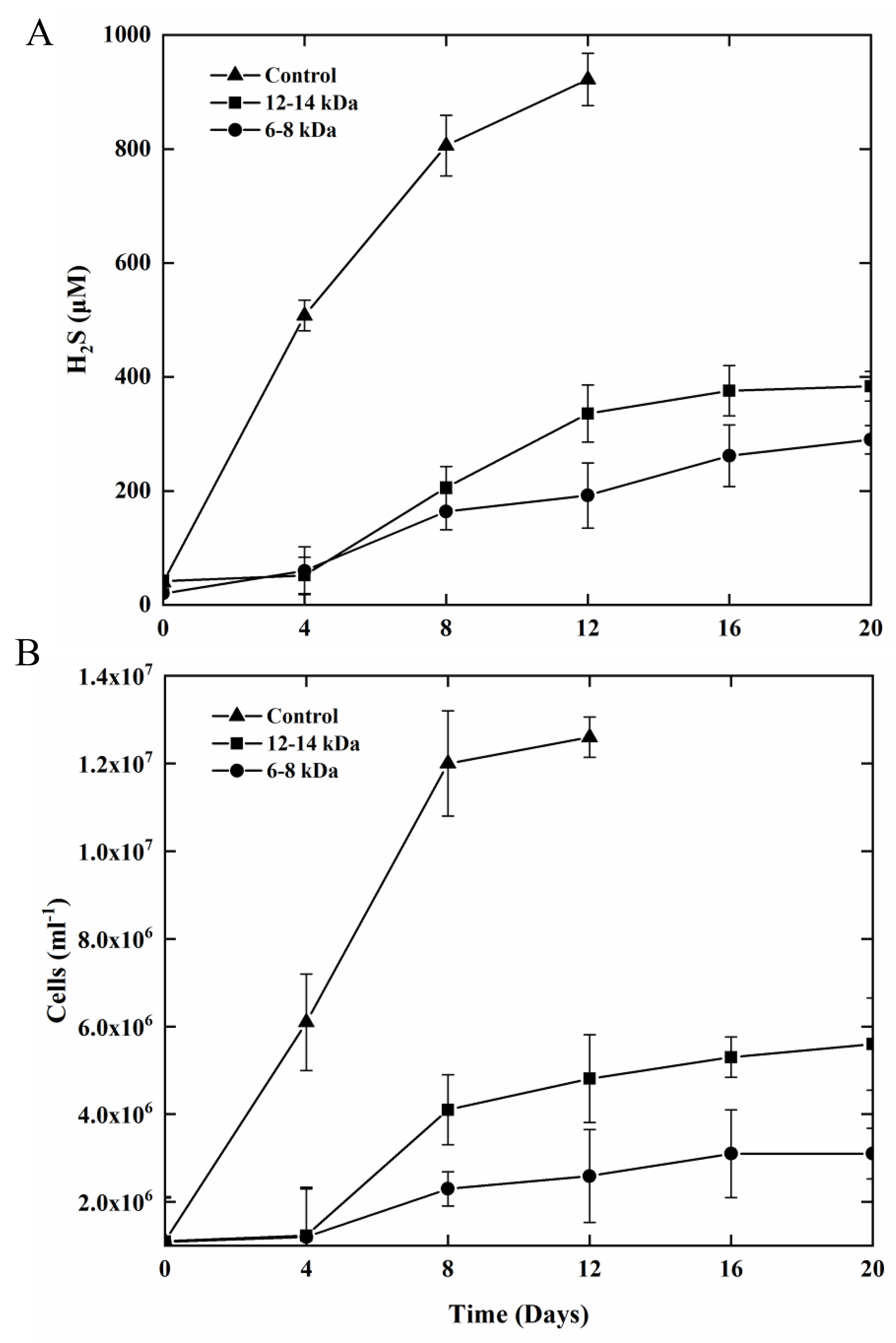

Supplement: FIG S4 [file msystems.00954-22-s0004.tif]
