# Supplementary material for: Disproportionation of Inorganic Sulfur Compounds by Mesophilic Chemolithoautotrophic Campylobacterota
Source: mSystems. 2022 Dec 21;8(1):e00954-22. doi: 10.1128/msystems.00954-22 (PMC9948710; doi:10.1128/msystems.00954-22)
Supplement: TABLE S1 [file msystems.00954-22-s0006.docx]

**Table S1** **Number of ASVs and alpha diversity indices about microbial communities.**

| **Sample** | **Total number of sequences** | **ASVs** | **Chao1** | **Shannon** |
| --- | --- | --- | --- | --- |
| ST0126 | 51001 | 7 | 7 | 0.18 |
| ST0104 | 46449 | 75 | 75 | 1.19 |
| ST0121 | 58601 | 59 | 59 | 2.10 |
| ST0116 | 45212 | 9 | 9 | 0.20 |
| ST0258 | 46592 | 35 | 35 | 0.92 |
| ST0246 | 54966 | 16 | 16 | 0.79 |
