# Supplementary material for: Disproportionation of Inorganic Sulfur Compounds by Mesophilic Chemolithoautotrophic Campylobacterota
Source: mSystems. 2022 Dec 21;8(1):e00954-22. doi: 10.1128/msystems.00954-22 (PMC9948710; doi:10.1128/msystems.00954-22)
Supplement: TEXT S1 [file msystems.00954-22-s0009.doc]

**Supplementary Information**

**Disproportionation of inorganic sulfur compounds by mesophilic chemolithoautotrophic *Campylobacterota***

Shasha Wang^1^, Lijing Jiang^1^*, Shaobin Xie^1^, Karine Alain^3^, Zhaodi Wang^1^, Jun Wang^1^ Delin Liu ^1^ and Zongze Shao^1, 2^*

^1^ Key Laboratory of Marine Genetic Resources, Third Institute of Oceanography, Ministry of Natural Resources of China; State Key Laboratory Breeding Base of Marine Genetic Resources; Fujian Key Laboratory of Marine Genetic Resources; Sino-French Laboratory of Deep-Sea Microbiology (MicrobSea), Xiamen 361005, PR China

^2^ Southern Marine Science and Engineering Guangdong Laboratory (Zhuhai), Zhuhai 519000, PR China

^3^ CNRS, Univ Brest, Ifremer, Unité Biologie et Ecologie des Ecosystèmes marins Profonds BEEP, UMR 6197, IRP 1211 MicrobSea, IUEM, Rue Dumont d’Urville, F-29280 Plouzané, France

*Corresponding author:

Zongze Shao, shaozz@163.com; Lijing Jiang, jianglijing@tio.org.cn

**Running title**: Sulfur disproportionation by *Campylobacterota.*

**Supplementary methods**

**The enrichment media**

MMJS liquid medium was a modified artificial seawater medium (MMJ) supplemented with elemental sulfur (Sigma-Aldrich, 1% w/v) as the sole energy substrate under the gas phase of 80% N_2_/20% CO_2_ (200 kPa), which is often used for the isolation of chemolithoautotrophic sulfur oxidizing bacteria [1]. MMJ medium consisted of the following components (l^-1^ MJ synthetic seawater): 30 g NaCl, 0.25 g NH_4_Cl, 0.33 g KCl, 0.14 g CaCl_2_·2H_2_O, 4.18 g MgCl_2_·6H_2_O, 0.14 g K_2_HPO_4_, 1 g NaHCO_3_, 0.5 mg NiCl_2_·6H_2_O, 0.5 mg Na_2_SeO_3_·2H_2_O, l ml Wolfe’s vitamins and 10 ml trace element solution [1]. All medium components, with the exception of NaHCO_3_, trace elements and vitamins, were boiled for less than 5 minutes under a gas stream of 100% N_2_ to remove dissolved oxygen. The insoluble elemental sulfur was in form of suspended grains, which was sterilized twice by autoclaving at 105℃ for 30 min in advance, and was added into MMJ medium by a sterilized spoon in an anaerobic glove box.

**Morphological and chemical characterization of iron-sulfides particles**

For SEM analysis, the morphological change­s on the surface of the iron-sulfides particles and the interaction of strain-particles were observed. The black suspension was collected using polycarbonate filters (Merck Millipore, pore size 3.0 µm), rinsed three times with deionized water and vacuum-dried under N_2_ atmosphere [2]. The samples were then observed by SEM at 10 kV and an EDS equipment used at an accelerating voltage of 15 keV for 30 s. The non-inoculated thiosulfate and elemental sulfur-disproportionating media were used as controls. To detect the crystalline mineral phases of the added FeOOH and the formed Fe-sulfides, all samples were subjected to XRD. The particles were centrifuged, washed and vacuum-dried. X-ray diffraction patterns of samples were obtained by a PANalytical X'Pert PRO X-ray diffractometer (radius: 240.0 mm). Incident X-ray radiation was produced from a line focused PW3373/10 Cu X-ray tube, operating at 40 kV and 40 mA with Cu Kα radiation of 1.54 Å. The scan step size and time per step were 0.03° and 10.16s, respectively.

**Supplementary results**

**Characterization of Fe-sulfide particles produced in cultures of sulfur disproportionating strains**

With thiosulfate, irregular clusters of nanoparticles were observed by SEM. In addition, a biofilm layer was observed on the surface of mineral particles (Fig. S3B). In the control, the standard fluffy, gridded shape of the added ferrihydrite was observed (Fig. S3A), and matched the morphology of the chemically synthesized ferrihydrite nanoparticles observed previously [3]. Bacteria were observed on the surface of iron sulfide particles (Fig. S3C). With elemental sulfur, obvious dents on the surface of elemental sulfur were observed (Fig. S3E and F) compared with the control (Fig. S3D), and the cells appeared to be attached to elemental sulfur and directly associated with the nanoparticles (Fig. S3F). From the EDS analysis, iron and sulfur represented the major elements of the nanoparticles (Fig. S3G and H).

The chemical composition of iron-sulfides particles was further examined with X-ray diffraction (XRD). With thiosulfate, the XRD patterns showed unique diffraction peaks at 2θ=17.52^o^, 30.14^o^, 38.92^o^ and 50.43^o^ (Fig. S3I), which are the characteristic peaks of mackinawite (FeS, PDF 15-0037). In addition, greigite (Fe_3_S_4_, PDF 16-0713) and marcasite (FeS_2_, PDF 37-0475) were also present with weakly distinguishable peaks (Fig. S3I). These minerals were not found in the negative controls. Thus, the greyish-black precipitate formed during thiosulfate disproportionation was mainly composed of FeS and possibly of Fe_3_S_4_ and FeS_2_. With elemental sulfur, three prominent peaks were clearly distinguishable. In details, peaks with 2θ values of 33.12^o^, 37.12^o^ and 40.78^o^, corresponding to the (200), (210) and (211) crystal planes of FeS_2_ nanoparticles (PDF 42-1340), were found (Fig. S3J). In addition, two diffraction peaks at 2θ = 28.45^o^ and 33.12^o^ were observed, which are the characteristic peaks of iron sulfides (FeS, PDF 23-1123) (Fig. S3J). These results showed that amorphous ferrihydrite during S^0^ disproportionation was converted to black Fe(II)-containing precipitates, such as FeS_2_ and FeS.

**Reference:**

1. Takai K, Inagaki F, Nakagawa S, Hirayama H, Nunoura T, Sako Y, Nealson KH, Horikoshi K. 2003. Isolation and phylogenetic diversity of members of previously uncultivated epsilon-Proteobacteria in deep-sea hydrothermal fields. FEMS Microbiol Lett 218:167-74.

1. Huo YC, Li WW, Chen CB, Li CX, Zeng R, Lau TC, Huang TY. 2016. Biogenic FeS accelerates reductive dechlorination of carbon tetrachloride by *Shewanella putrefaciens* CN32. Enzyme Microb Technol 95:236-241.
2. Cazacu N, Chilom CG, Iftimie S, Balasoiu M, Ladygina VP, Stolyar SV, Orelovich OL, Kovalev YS, Rogachev AV. 2022. Biogenic ferrihydrite nanoparticles produced by *Klebsiella oxytoca*: characterization, physicochemical properties and Bovine Serum Albumin interactions. Nanomaterials 12:249.
