# Supplementary material for: Disproportionation of Inorganic Sulfur Compounds by Mesophilic Chemolithoautotrophic Campylobacterota
Source: mSystems. 2022 Dec 21;8(1):e00954-22. doi: 10.1128/msystems.00954-22 (PMC9948710; doi:10.1128/msystems.00954-22)
Supplement: TABLE S2 [file msystems.00954-22-s0007.docx]

Table S2 **Detail information on the sampling sites.**

| **Sample name** | **Station sites** | **Longitude (E)** | **Latitude (N)** | **Depth (m)** | **Collection time** | **Sample type** |
| --- | --- | --- | --- | --- | --- | --- |
| **ST0126** | **57II-CR-ST01-26** | **60^o^68’** | **6^o^56’** | **–2975** | **2018.09.11** | **Hydrothermal plume** |
| **ST0104** | **57II-CR-ST01-04** | **60^o^67’** | **6^o^55’** | **–2968** | **2018.09.11** | **Hydrothermal plume** |
| **ST0121** | **57II-CR-ST01-21** | **60^o^63’** | **6^o^51’** | **–2970** | **2018.09.15** | **Hydrothermal plume** |
| **ST0116** | **57II-CR-ST01-16** | **60^o^63’** | **6^o^51’** | **–2942** | **2018.09.15** | **Hydrothermal plume** |
| **ST0258** | **57II-CR-ST02-58** | **60^o^58’** | **6^o^46’** | **–2875** | **2018.09.18** | **Hydrothermal plume** |
| **ST0246** | **57II-CR-ST02-46** | **60^o^58’** | **6^o^46’** | **–2879** | **2018.09.20** | **Hydrothermal plume** |
| ST0223 | 57II-CR-ST02-63 | 60^o^59’ | 6^o^47’ | **–**2862 | 2018.09.20 | Hydrothermal plume |
| ST0224 | 57II-CR-ST02-63 | 60^o^59’ | 6^o^47’ | **–**2843 | 2018.09.26 | Hydrothermal plume |
| ST0228 | 57II-CR-ST02-21 | 60^o^59’ | 6^o^47’ | **–**2840 | 2018.09.26 | Hydrothermal plume |

The samples in bold font are the ones for which we got positive enrichment cultures of microbial sulfur disproportionation from deep-sea hydrothermal vent plume.
